# The crucial roles of ICT, renewable energy sources, industrialization, and institutional quality in achieving environmental sustainability in BRICS

**DOI:** 10.1007/s11356-024-33479-4

**Published:** 2024-05-08

**Authors:** Charles Shaaba Saba, Charles Raoul Tchuinkam Djemo, Nicholas Ngepah

**Affiliations:** School of Economics, College of Business and Economics, University of Johannesburg, Auckland Park Kingsway Campus, PO Box 524, Johannesburg, Auckland Park South Africa

**Keywords:** Information and communications technology (ICT), Renewable energy sources, Industrialization, Environmental sustainability, Institutional quality, CS-ARDL panel analysis, BRICS, C33, O14, O33, Q2, Q56

## Abstract

The BRICS countries—Brazil, Russia, India, China, and South Africa—are committed to achieving United Nations Sustainable Development Goal 13, which focuses on mitigating climate change. To attain this goal, it is crucial to emphasize the significance of ICT, renewable energy sources, industrialization, and institutional quality. This study contributes to the literature by examining the potential role of these factors in environmental sustainability in the BRICS economies from 2000 to 2021, utilizing cross-sectional augmented autoregressive distributed lag (CS-ARDL) estimation and other novel econometric techniques. Accordingly, the study suggests that BRICS governments and policymakers prioritize the use of ICT in the industrial and institutional sectors to achieve faster environmental sustainability in the short-run, as per the CS-ARDL results. However, the study advises caution in the long-term as the interaction between ICT and renewable energy sources, industrialization, and institutional quality may not favour environmental quality. Although the renewable energy sources interaction with ICT may not yield immediate progress, strong measures need to be taken to ensure that short-term gains are not nullified. In conclusion, the study highlights the potential of ICT, renewable energy sources, industrialization, and institutional quality in achieving environmental sustainability in the BRICS countries, while recommending cautious measures in the long run to safeguard the progress made.

## Introduction

**Fig. 1:**
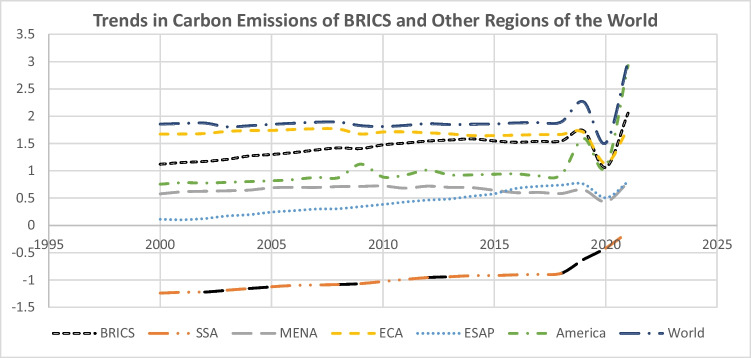
Average trend in CO_2_ emissions across regions according to World Bank classification (Sub-Saharan Africa (SSA), Middle East and North Africa (MENA), Europe & Central Asia (ECA), East & South Asia and the Pacific (ESAP) and America) and BRICS from 2000–2021. **Source**: Author’s computation using World Development Indicators (WDI) data

This study’s position of inquiry is driven by four primary considerations, namely: (i) information and communication technology (ICT) diffusion potentials in all the sectors of Brazil, Russia, India, China and South Africa (BRICS) economies; (ii) the challenge of achieving Sustainable Development Goals (SDGs) 13 and 7 (i.e. SDGs 13 (combating climate change) and 7 (affordable and clean energy)) in BRICS countries; (iii) the vital role of industrialization, renewable energy sources and institutional quality in either propulsive or mitigating environmental pollution (for example, carbon dioxide (CO_2_) emissions); and (iv) there are research gaps in the empirical literature on the environment that require attention. According to the International Energy Agency (IEA) report from 2022, when compared to other regions of the world, the combined emissions per capita of the BRICS economies are among the highest, and they contribute more than three percent of global energy-related CO_2_ emissions to date (see the graphical trends in Fig. 1 as an evidence). The continuous use of fossil fuels by BRICS countries poses a serious threat to environment quality. Therefore, it has become important to explore all possible avenues/measures to combat climate change and its impact in BRICS.

According to previous ICT studies, there is a significant amount of opportunity for ICT diffusion in BRICS when contrasted with more developed regions such as in Europe, North America etc., where ICT diffusion has been well established (see Pénard et al. 2012; Mirza et al. 2020). This is because policymakers can utilize ICT to address obvious policy concerns around sustainable development (for example, environmental pollution and climate change/global warming) given the potential role it plays in every society.

Since environmental sustainability/combating climate change became top policy agenda after the post-2015 development era, therefore, we should be worried about environmental sustainability in BRICS economies for at least four key reasons:

- the BRICS countries have achieved high levels of economic growth and are among the fastest growing economies globally, particularly when compared to other developing countries. For example, according to the World Bank, the BRICS countries collectively grew at an average annual rate of 5.8% between 2000 and 2010, compared to 2.6% for high-income countries even with all the fluctuations that took place;
- the continuous crisis/challenges of investing, accessing and utilizing renewable energy in this era of achieving sustainable development;
- the countries have not reached perfection in energy management; and
- the post-2015 sustainable development agenda's main concerns center on the harmful effects of climate change. Huxster et al. (2015) assert that the unsustainable use of fossil fuels is the root cause of these issues. In addition, Shah et al. (2022) argued that Africa would experience the worst effects of climate change.

The role of ICT in managing/promoting environmental resources/sustainability sparks a new discussion among policymakers on how to advance technology and reduce high mass CO_2_ emissions globally. On the one hand, ICT penetration have the potential to contribute significantly to environmental sustainability (Khan et al. 2022a, b). This is because ICT can be used to promote sustainable practices, monitor and manage natural resources, reduce energy consumption, and mitigate the impacts of climate change (*Inter alia*: Yap 2011; Titifanue et al. 2017; Qureshi 2019; Lahouel et al. 2021; Amari et al. 2022).

Some of the ways in which ICT can contribute to environmental sustainability across the globe as identified in the literature include: electronic waste (e-waste) management through proper disposal and recycling of electronic devices (Nnorom and Osibanjo 2008; Nivedha and Sutha 2020; Shahabuddin et al. 2022); promoting the use of renewable energy sources such as solar, wind, and hydro power (Saba and Biyase 2022; Chang et al. 2022); promoting sustainable agricultural practices such as precision farming, which reduces the use of pesticides and fertilizers, and to monitor and manage water resources for irrigation (smart agriculture) (Meena et al. 2012; Adamides et al 2020); managing natural disasters such as floods, droughts, and wildfires by providing early warning systems, real-time monitoring of affected areas, and coordinating relief efforts (disaster management) (Yap 2011; Md Hassan 2015; Cacciotti et al. 2021); and promoting sustainable transport systems such as public transport, carpooling, and cycling, reducing the use of fossil fuels and air pollution (*Inter alia*: Agarwal and Alam 2018; Gössling 2018; Chatti 2021). Even though ICT have the potential to also contribute significantly to environmental sustainability in BRICS countries, however, for it to be effective in promoting environmental sustainability, it must be integrated into policies and strategies aimed at achieving sustainable development in the economic bloc. On the other hand, ICT could play a significant role in carbon emissions due to its energy consumption, which contributes to greenhouse gas emissions. The use of ICTs, including smartphones, laptops, servers, and data centers, requires electricity to operate, which is mostly generated from non-renewable sources of energy. As a result, the ICT sector could contribute to carbon emissions. According to a report by the International Energy Agency (IEA) (2020), the ICT sector was responsible for 1.4% of global carbon emissions in 2020. This figure is projected to increase to 3.5% by 2025 if no action is taken to reduce the energy consumption of ICTs. The energy consumption of ICT infrastructure, such as data centers, servers, and network equipment, is a major contributor to carbon emissions and also the production, transportation, and disposal of ICT devices also contribute to carbon emissions (Higón et al. 2017).

Recent studies on ICT and environmental sustainability across various global regions underscore ICT's potential to drive sustainable development and tackle environmental issues. Reports from international organizations like the World Bank (2021), United Nations Development Programme (UNDP) and the International Finance Corporation (IFC) (2021) and African Development Bank (AfDB) (2021) discuss ICT's role in enhancing climate resilience in different countries. These reports highlight ICT solutions such as weather forecasting and early warning systems that aid communities in adapting to climate change. They also stress the importance of rigorous empirical research to guide policies and regulations supporting the implementation of these solutions.

Several studies have examined the potential of renewable energy sources (RESS) to contribute to environmental sustainability both at panel and country-specific levels. For example, a study in South Africa found that the deployment of wind energy could lead to significant emissions reductions and improve air quality (Mubiru et al. 2019). In a study on BRICS economies, Shah et al. (2022) explore the link between renewable energy, agriculture, GDP, and CO_2_ emissions. They conclude that renewable energy can influence the Environmental Kuznets Curve hypothesis. Additionally, Bano et al. (2022) propose a policy framework for these economies to shift towards renewable energy, supporting green tourism development. The study explores the role of aging, industrial innovations, and ICT on tourism and renewable energy consumption and finds that aging, industrial innovations, and ICT play a significant role in promoting renewable energy and sustainable tourism development in BRICS economies. Industrialization and CO_2_ emissions are two important issues in BRICS given the levels of industrial development in the countries. The recent study of Voumik and Sultana (2022) examines the impact of industrialization, urbanization, electrification, and renewable energy on the environment in BRICS countries. The study finds that these factors have a significant effect on protecting water, land, and forest resources while lowering carbon emissions.

Institutional quality is widely recognized as a critical factor in promoting sustainable development and reducing greenhouse gas emissions. This is because strong institutions can create an enabling environment for the adoption of environmentally friendly policies and practices, and promote accountability and transparency in environmental governance. Several studies, including those of Zhang et al. (2019), Gyamfi and Zhou (2020), Olaniran and Adeyemo (2020), Jiang et al. (2022) among others, have investigated the relationship between institutional quality and CO_2_ emissions. These studies have highlighted the fact that strengthening institutions is an ongoing task that countries must continue to undertake to achieve meaningful progress in society. This underscores the importance of examining institutional quality-ICT induced impact on environmental sustainability in the BRICS economy given that institutions also make use of ICT infrastructures/services for their day-today activities.

The central research question that previous studies have not addressed, and which we aim to explore, is how BRICS nations can achieve environmental sustainability by utilizing their sustainable policy tools in the areas of ICT, renewable energy sources, industrialization, and institutional quality. The specific sub-research questions are as follows: does the interaction between ICT and renewable energy sources impact CO_2_ emissions in BRICS countries?; does the interaction between ICT and industrialization affect CO_2_ emissions in BRICS countries?; and does the interaction between ICT and institutional quality influence CO_2_ emissions in BRICS countries? These questions guide the study's objectives and contributions. The overarching goal is to examine how ICT complements industrialization, renewable energy sources, and institutional quality to influence environmental sustainability in the BRICS economies from 2000 to 2021.

Our research on BRICS contributes to the existing literature in several ways compared to previous empirical studies. Firstly, it investigates the impact of the interaction between ICT and industrialization on CO_2_ emissions. Secondly, it examines the impact of the interaction between ICT and renewable energy sources on CO_2_ emissions. Thirdly, it investigates the impact of the interaction between ICT and institutional quality on CO_2_ emissions. These were investigated and the results were obtained within the context of short and long run estimates.

This study holds both academic and policy relevance due to the objectives outlined above. The primary theoretical contribution of this study is that it demonstrates how a modified Cobb–Douglas production function (Cobb and Douglas 1928), following previous empirical studies, can be used to investigate the objectives of this study. Under the research topic for this study, our findings suggested mitigation methods from a variable interaction approach at the panel level. We also take into account the issues of homogeneity and cross-sectional dependence (CD) in the series under the unit root and cointegration techniques. Most panel series in the literature assume that the residual terms are unrelated, which ignores the issues of CD between countries. Accounting for the issues of CD in the series is essential as BRICS countries possess varying levels of economic tiles. We applied the Dumitrescu and Hurlin (2012) panel causality technique that considers heterogeneity among countries. Additionally, to the best of our knowledge, this is the first study to simultaneously employ the novel Cross-Sectional Autoregressive-Distributed Lag (hereafter, CS-ARDL) technique proposed by Chudik and Pesaran (2015), as well as Augmented Mean Group (henceforth, AMG) and dynamic Common Correlated Effects Mean Group (CCEMG) estimation techniques, to analyze panel data from 2000–2021. While we used the AMG and CCEMG estimators to validate our results, we primarily relied on the CS-ARDL approach, given its advantages over the others. The CS-ARDL method has several advantages over other panel data estimators. It yields robust results in the presence of cross-sectional dependence and can be applied to series with different orders of integration, including *I(0*), *I(1)*, or both. Additionally, it produces accurate results in cases of weak exogeneity and allows for both pooled, mean group, and pooled-mean group estimates based on the homogeneity or heterogeneity of slope coefficients.

Based on the CS-ARDL results, which were further validated by the use of AMG and CCEMG approaches, this study recommends that BRICS governments and policymakers prioritize the use of ICT in the industrial and institutional sectors to achieve faster environmental sustainability in the short run. While the study advises caution in the long term as the interaction between ICT and renewable energy sources, industrialization, and institutional quality may not favour environmental quality. In conclusion, the study highlights the potential of ICT, renewable energy sources, industrialization, and institutional quality in achieving environmental sustainability in the BRICS countries, while recommending cautious measures in the long run to safeguard the progress made.

The organization of this paper is as follows: Sect. 2 presents the literature review. Section 3 presents the methodology and data. Results from the empirical analysis are presented and discussed in Sect. 4, while policy implications are found in Sect. 5. Finally, Sect. 6 concludes the study.

## Literature review, theoretical framework and hypothesis development

### Theoretical framework and hypothesis development

The existing literature on the value of ICT at the country level primarily indicates that ICT investment positively impacts productivity in developed countries, but not in developing ones. However, with continued investment in ICT infrastructure, it is crucial to reassess its benefits in developing countries. Dedrick et al. (2013) explored this by analyzing data from 45 countries between 1994–2007, comparing it to the 1985–1993 period. They found that upper-income developing countries experienced significant productivity gains from ICT investment in the more recent period, with the effects of ICT on productivity being influenced by factors such as human resources and openness to foreign investment. Beyond productivity, ICT also holds potential for environmental sustainability, such as reducing emissions through smarter cities and optimizing production processes. Watson et al. (2010) highlight ICT's transformative ability to foster an environmentally sustainable society. This broadens the scope of ICT value research to include environmental aspects, aligning with the triple bottom line (TBL) concept that encompasses economic, environmental, and social dimensions of value.

The study is grounded in the Environmental Kuznets Curve (EKC) theory, which suggests that environmental degradation initially worsens with economic development, peaks at a certain point, and then improves as growth continues. The EKC framework posits that technological advancements and energy conservation can enhance environmental sustainability. Information and Communication Technologies (ICTs) are identified as a key factor in promoting environmental sustainability, impacting it in three ways: directly through consumption, indirectly through substitution, and systemically through rebound effects. The direct negative impacts of ICTs include the use of non-renewable energy in manufacturing ICT equipment and the disposal of e-waste (Park et al. 2018). However, ICTs can also positively influence environmental sustainability through green ICT initiatives, smarter cities, digital education, e-health, e-commerce, and virtual meetings (Shabani and Shahnazi 2019). Nonetheless, energy-intensive ICT activities can increase energy consumption, and the rebound effect—where the increased efficiency of ICT goods and services leads to greater usage—can exacerbate pollution (Danish et al. 2018a, b). Additionally, the expansion of business activities and communication networks facilitated by ICT can negatively affect environmental quality (Ozcan and Apergis 2018).

The relationship between renewable energy sources and environmental sustainability is a critical area of study in the quest for sustainable development. Renewable energy sources, such as solar, wind, hydro, and biomass, are considered vital for achieving environmental sustainability due to their lower environmental impact compared to fossil fuels. Renewable energy sources produce significantly lower greenhouse gas emissions compared to fossil fuels, thereby mitigating climate change. For example, solar and wind energy have near-zero emissions during operation (IPCC 2021). Unlike fossil fuels, renewable energy sources are abundant and replenishable, reducing the strain on finite natural resources and ensuring long-term energy security (Goldemberg and Lucon 2009). Renewable energy technologies generate minimal air and water pollutants, improving air quality and reducing health risks associated with pollution (Jacobson et al. 2018). By reducing habitat destruction and pollution, renewable energy sources can help protect biodiversity and ecosystems (Sovacool and Dworkin 2014). The deployment of renewable energy can drive economic development through job creation, technology innovation, and energy access in remote areas (IRENA 2020). However, the transition to renewable energy is not without challenges, such as variability, storage, and the need for grid integration. Addressing these challenges requires technological advancements, policy support, and investment in renewable energy infrastructure.

The success of environmental policy measures is heavily influenced by the effectiveness of the institutions responsible for their implementation. Strong institutions can enforce laws that protect the environment and promote sustainable growth (Azam et al. 2021a, b). However, political instability can undermine the government's ability to enforce pollution control policies, and political preferences on environmental issues can shift (Mrabet et al. 2021). Furthermore, weak institutions may be exploited by rent-seeking investors, leading to the failure of environmental policies (Baloch and Wang 2019).

Theoretical literature suggests that industrial development can impact environmental degradation. According to the theory of ecological modernization, industrial activities are part of a social transformation process that promotes modernization but can also lead to environmental issues. However, the negative environmental effects of industrial development may lessen due to technological advancement, urbanization, and a shift from a manufacturing-based economy to a service-based economy (Mol and Spaargaren 2000). The urban environmental transition theory similarly suggests that while manufacturing expansion can increase wealth, it may also intensify environmental pollution. Nevertheless, as a society becomes wealthier, pollution may decrease due to technological improvements or changes in the economic sectors' composition or structure (Hussain and Zhou 2022). Ehigiamusoe (2023) argued that industrial activities can emit carbon and other greenhouse gases, while Wen et al. (2014) noted that industrial development might not worsen carbon emissions if there is environmental awareness and the adoption of energy-efficient technologies. Drawing from the theoretical discussion above, this study examines the formulated hypotheses as follows:

- $Hypothesis {H}_{aii}:$ *ICT development, renewable energy sources, institutional quality and industrialization causes environmental sustainability path in BRICS.*
- $Hypothesis {H}_{bii}:$ *ICT development, renewable energy sources, institutional quality and industrialization promotes environmental sustainability path in BRICS.*
- $Hypothesis {H}_{cii}:$ *The joint impact between ICT development and renewable energy sources, promotes environmental sustainability path in BRICS.*
- $Hypothesis {H}_{dii}:$ *The joint impact of ICT development and institutional quality promotes environmental sustainability path in BRICS.*
- $Hypothesis {H}_{eii}:$ *The joint impact of ICT development and industrialization promotes environmental sustainability path in BRICS.*

### Empirical literature

Global warming, precipitation variability, and significant climate changes pose a serious threat to sustainable development in BRICS countries, particularly affecting agricultural production and food and water supplies. This can directly impact investor behavior, financial markets, welfare, the environment, and economic growth. The increasing trends of extreme temperatures and rainfall in BRICS countries heighten the risks and uncertainties of environmental, climatic, and economic instability. Governments, farmers, and scholars are increasingly concerned about climate change risks to the economy, and adaptation strategies have become a crucial focus for researchers and policymakers. Understanding the nexus between CO2 emissions, renewable energy sources, ICT, and institutional quality is vital for the economic and environmental sustainability of BRICS countries. Some literature has explored the connections between pollution, agriculture, and economic development, as well as agriculture, energy consumption, and industrialization in developing and emerging countries. This research aims to provide policymakers with the necessary tools to mitigate climate risks and implement adaptation strategies.

Despite ongoing empirical research in environment and energy, climate change challenges keep the topic relevant, especially for developing nations. Many studies have examined the link between environmental and macroeconomic variables, such as agricultural performance and economic growth, often finding a bidirectional causal relationship between pollution and agricultural value added in regions like the EU and North Africa. (see for example, Bekun et al. 2019; Jebli and Youssef 2017 among others). In contrast, Asumadu-Sarkodie and Owusu (2016) sought to investigate the interconnections among CO_2_ emissions, electricity consumption, industrialization, and economic development in Benin. The results of the long-run analysis show the existence of a positive relationship between industrialization and CO_2_ emissions. In a study focused on Zambia’s economy, Phiri et al. (2021) highlighted the influence of agricultural practices on pollution. They also examined the relationship between agriculturalization, industrialization, and economic growth, finding that these factors are interrelated and tend to stabilize over time. However, their research is limited to certain African countries and does not explore other factors crucial for a sustainable environment, such as the interplay between ICT, renewable energy sources, and institutional quality.

For instance, limited empirical studies (Karim et al. 2022; Avom et al. 2020) focusing on the impact of institutional quality and other environmental factors in Sub-Saharan African countries suggest that strong institutional quality significantly reduces environmental pollution. Conversely, Acheampong et al. (2021) contend that there is no causal relationship between institutional quality and economic development. It is essential to categorize existing research on environmental sustainability into three distinct groups for a clearer understanding. Primarily, studies have focused on evaluating the role of the financial sector and its development (FD), information systems (ICTs), and renewable energy consumption (REC) as key factors in environmental pollution. For instance, Lahouel et al. (2021) examined Tunisia, Shabani and Shahnazi (2019) looked at Iran, Higón et al. (2017) covered selected developing and 26 industrialized economies, N’dri et al. (2021) focused on developing countries, Sahoo et al. (2021) studied India, Gyamfi et al. (2022) analyzed G7 countries, and Shobande and Ogbeifun (2022) investigated 24 OECD countries. The findings on the impact of ICTs on CO_2_ emissions or environmental degradation remain inconclusive, with Shabani and Shahnazi (2019) noting a unidirectional causal relationship between ICT, economic development, and energy consumption with CO_2_ emissions in the long term.

Conversely, Lahouel et al. (2021) observed a nonlinear relationship between CO_2_ emissions and ICT, suggesting that ICT plays a crucial role in mitigating climate risk and promoting economic growth. Higón et al. (2017) explored the connection between environmental variables such as CO_2_ emissions, economic growth (GDP), and ICT, finding a positive correlation between pollution and ICT, indicated by an inverted U-shaped curve. Similarly, N’dri et al. (2021) found that ICTs can reduce pollution in the long term. Sahoo et al. (2021) extended their analysis to India, including financial development and electricity alongside economic growth and CO_2_ emissions, and argued that ICT negatively affects environmental sustainability while monetary expansion is inversely related to CO_2_ emissions. Shobande and Ogbeifun (2022) also assert that ICT promotes ecological sustainability, highlighting the contribution of ICT and financial development to ecosystem sustainability.

A secondary group of research studies delves into the impact of institutional quality on environmental quality. Riti et al. (2021) investigated this relationship in ten countries with high press freedom, while Goel et al. (2013) focused on MENA economies. Chien et al. (2021) and Hussain and Dogan (2021) directed their studies towards BRICS economies, and Karim et al. (2022) examined 30 Sub-Saharan African countries. These studies collectively aim to understand the link between the quality of institutions and environmental outcomes in various global regions. Goel et al. (2013) examined the effects of corruption and the informal sector on environmental pollution, discovering that high corruption levels and the presence of a shadow sector can obscure actual CO_2_ emissions. This underscores the importance of institutional management in mitigating environmental pollution.

Similarly, Riti et al. (2021) assessed the role of press freedom in influencing environmental degradation across ten countries, finding that greater press freedom is likely to reduce environmental degradation. Karim et al. (2022) evaluated the impact of institutional quality on CO_2_ emissions in Sub-Saharan Africa and found that measures like corruption control, legislative quality, and rule of law significantly lower CO_2_ emissions in the region. Furthermore, they find the existence of two-way causality between all institutional quality indices and CO_2_ emissions. Henceforth, effective governance policies and regulations mitigate climate risk. Aligning the result with previous papers concentrating on the nexus between institutional quality and ecological pollution, Hussian and Dogan (2021) conclude that institutional quality and environmental-related technology negatively impact the environmental footprint in the BRICS economies. Jebli et al. (2020) investigated the nexus between CO_2_ emissions, renewable energy consumption and economic growth using the generalized method of moments model. This study applied the econometric method to data from 1990 to 2015 for 102 countries classified by income level. The findings reveal a causality link between renewable energy consumption and other variables in all countries under study, decreasing CO_2_ emissions.

Radmehr et al. (2021) focused on the EU’s regional economic zone, using the generalized spatial two-stage least square (GS2SLS) method to find that financial performance and renewable energy consumption have a unidirectional causal relationship and are positively correlated across countries. They also found a bidirectional link between CO_2_ emissions and economic growth and renewable energy consumption. Acheampong et al. (2021) used a different approach, the GMM-based panel vector autoregressive technique, to study the intertemporal causality between economic growth, renewable energy, institutional quality, and CO_2_ emissions. They found a bidirectional causality between renewable energy consumption and economic growth in the long run but no causality between economic growth and institutional quality. This contrasts with findings from Gyamfi et al. (2022), Riti et al. (2021), and Goel et al. (2013), who suggested that institutional quality can lead to environmental sustainability.

Furthermore, several studies have investigated the relationship between industrialization and environmental degradation, particularly focusing on CO_2_ emissions. For example, an analysis of 99 countries across different income levels found that industrialization increases CO_2_ emissions in all groups. Li and Lin (2015) confirmed this detrimental impact using data from 76 countries, while Sohag et al. (2017) reported similar findings for 86 nations. Opoku and Aluko (2021) employed quantile regression to examine the impact of industrialization on the ecological footprint in 37 African countries, finding mixed effects across different quantiles. Kahouli et al. (2022) identified a long-run unidirectional causal relationship from industrialization to CO_2_ emissions in Saudi Arabia, with bidirectional causality in the short run. Similar unidirectional causality was reported by Nasir et al. (2021) in Australia, where Rahman and Alam (2022) also found bidirectional causality.

Usman and Balsalobre-Lorente (2022) observed that industrialization contributes to the ecological footprint in 10 newly industrialized countries. However, Naudé (2011) argued that industrialization could reduce emissions associated with agriculture by shifting labor to more energy-efficient and cleaner industrial sectors. Rafiq et al. (2016) found that industrialization reduces CO_2_ emissions in high-income countries but has an insignificant effect in low- and middle-income countries. Zhou et al. (2013) discovered that industrial adjustment can mitigate CO_2_ emissions in China, while Lin et al. (2015) and Ehigiamusoe (2023) found no adverse effects of industrialization on CO_2_ emissions in Nigeria and ASEAN + China, respectively.

Renewable energy is increasingly recognized as a means to promote environmental and ecological sustainability alongside economic growth (Nazir 2023). However, empirical studies exploring the relationship between renewable energy consumption and industrial growth are limited. Dey et al. (2023) and Van Hoang (2020) are among the few who have examined this relationship. Van Hoang (2020) found that in the United States, renewable energy consumption not only supports industrial production, but the type of energy also plays a significant role. Specifically, biomass energy had a more pronounced effect on industrial production compared to hydroelectric and geothermal energy, though its impact takes time to manifest. The study also identified a bidirectional causality between renewable energy consumption and industrialization.

Dey et al. (2023) analyzed the effects of various renewable energy sources, including biomass, solar, wind, bagasse, small hydropower (SHP), and waste, on sustainable industrialization in India. Using quantile regression, the study found that SHP and bagasse, in particular, contributed to increased industrial production. The research also revealed bidirectional non-linear causality between biomass, waste heat, and industrial production, and unidirectional non-linear causality with other energy sources. Mentel et al. (2022) investigated the relationship between industrialization and CO_2_ emissions in Central Asia and Europe, considering the moderating effect of renewable energy. Employing the two-step GMM method and data from 2000 to 2018, the study concluded that industrial development exacerbates CO_2_ emissions, while the moderating effect of renewable energy is negative.

Fossil fuels, which account for nearly one-third of global pollution, are considered one of the least sustainable energy sources and contribute significantly to environmental damage (Kumar and Majid 2022). Rapier (2020) notes that energy from natural resources fulfilled 84% of the total energy demand up to 2019, highlighting their role as major pollutants. To achieve environmental sustainability, transitioning to renewable energy consumption is essential as it can meet energy demands with fewer environmental impacts (He et al. 2023). Nasreen et al. (2017) emphasize that unsustainable energy sources like coal and fuel have serious environmental consequences. Conversely, renewable energy is key to reversing environmental damage and attaining environmental sustainability. Wada et al. (2021) argue that natural resources-powered fuels such as coal and gas increase pollution levels, whereas renewable energy offers a viable solution to this issue.

Iorember et al. (2020) find that renewable energy can reduce CO_2_ emissions and suggest that governments should develop policies promoting long-term renewable energy use. Achuo et al. (2022) note that energy efficiency through increased renewable energy can significantly reduce environmental pollution and achieve environmental sustainability. Asongu et al. (2020) highlight the dangers of continued fossil fuel use and advocate for a shift towards renewable energy. Liu et al. (2022) suggest that alternative energy can reduce pollution in thermal machines. Overall, renewable energy is crucial for promoting environmental sustainability.

Asumadu and Owusu (2016) explored the causal relationship in African countries, specifically in Benin's economy, using the ARDL approach. They discovered that a slight 1% increase in industrialization and electricity consumption leads to a rise in CO_2_ emissions. However, the paper did not assess the impact of ICT on CO_2_ emissions nor provided policies for sustainable environmental management. Sustainable development is a key agenda for the BRICS union, achievable through policies promoting ecological sustainability. Thus, it is essential to extend previous research to investigate the role of ICT in enhancing environmental sustainability in BRICS economies. In the light of the above previous literature, we further carried out a literature survey on the nexus between ICT, renewable energy sources, industrialization, institutional quality and CO_2_ emissions which is examined from four perspectives: ICT impact on CO_2_ emissions (both direct and indirect impacts); institutional quality-CO_2_ emissions nexus; renewable energy**-**CO_2_ emissions nexus; and industrialization-CO_2_ emissions nexus (see Table 1). The results reveal various scenarios and insights derived from these linkages.

**Table 1:** Literature survey on the nexus between ICT, renewable energy, industrialization, institutional quality and CO_2_ emissions

Authors & Period	Countries	Methodology	Findings
Godil et al. (2020), 1995Q1–2018Q4	Pakistan	QARDL	ICT, FDV decrease CO_2_ emissions and INSQTY increase CO_2_ emissions
Ahmed and le (2021), 1990–2017	ASEAN-6 countries	CUP-FM, CUP-BC	ICT decreases CO_2_ emissions
Khan et al. (2020), 1990–2017	Panel of 91 countries	POLS, FEM, SGMM	ICT decreases CO_2_ emissions: for the global panel and for developed countries ICT increases CO_2_ emissions: developing countries
Sana et al. (2021), 1990–2018	92 countries	POLS, FEM, SGMM	ICT decreases CO_2_ emissions GDP decrease CO_2_: full sample and developing countries
Weili et al. (2022), 2000–2019	Belt and Road countries	OLS, GMM and GLS	ICT increases CO_2_ emissions
Batool et al. (2022), 1985–2020	Developing countries of Asia	ARDL-PMG	ICT, FDV and GDP increase CO_2_ emissions
Park et al. (2018), 2001–2014	23 EU countries	PMG	ICT increases CO_2_ emissions
Tsimisaraka et al. (2023), 2004–2019	Top 10 emitter countries in the OBOR region	CS-ARDL	ICT increases CO_2_ emissions
Higón et al. (2017), 1995–2010	142 countries,	POLS	ICT and CO_2_ emissions exhibited an inverted-U shaped relationship
Ben lahouel et al. (2022), 1990–2019	16 MENA countries	PSTR	The relationship between ICT and CO_2_ emissions is nonlinear and negative, with higher levels of ICT associated with a decrease in CO_2_ emissions per capita
Shahnazi and Dehghan (2019), 2001–2015	Iran’s provinces	The dynamic spatial Durbin model	ICT and CO_2_ emissions: inverted-U shaped relationship
Salahuddin et al. (2016), 1985–2012	Australia	ARDL	ICT is not significant
Raheem et al. (2020), 1990–2014	G7 countries	MG	ICT decrease CO_2_ emissions ICT*FD: yields positive and significant coefficient ICT can amplify the effect of FDV on CO_2_ emissions
Danish et al. (2018a, b), 1990–2015	Next-11 countries	MG, AMG	ICT*FDV: increases CO_2_ emissions ICT*GDP: decreases CO_2_ emissions
Khan et al. (2022a, b), 1990–2019	BRICS	GLS, PCSE	ICT, RENW, decrease CO_2_ emissions GDP and FDV increase CO_2_ emissions ICT*RENW, ICT*DF, ICT*GDP decrease CO_2_ emissions
Abid (2017), 1990–2011	41 European Union (EU) and 58 Middle East and African (MEA) economies	System-GMM	The findings indicated that optimal economic development and, consequently, a reduction in CO_2_ emissions in specific countries depend on institutional quality
Salman et al., (2019a, b), 1990–2016	3 East Asian nations	DOLS and FMOLS	The conclusion drawn is that effectively organized and independent state institutions play a pivotal role in fostering economic growth and mitigating CO_2_ emissions
Zakaria & Bibi (2019), 1984–2015	South Asia	2SLS and GLS	INSQTY exert a notable negative influence on CO_2_ emissions
Le & Ozturk (2020), 1990–2014	47 developing countries	CCEMG, AMG, and DCCE	The results indicate that INSQTY is associated with an increase in CO_2_ emissions
Teng et al., (2021), 1985–2018	10 OECD economies	PMG	INSQTY positively influences environmental degradation
Boontome et al. (2017), 1971 − 2013	Thailand	GRC	Unidirectional causality from NREW to CO_2_; no causality between RENW and RGDP; no causality between RENW and CO_2_; no causality between RGDP and CO_2_
Lu (2017), 1990 − 2012	24-Asian countries	VECM	Unidirectional causality from RGDP to CO_2_; Bidirectional causality between RENW and CO_2_
Sebri and Ben-Salha (2014), 1971 − 2010	BRICS	ARDL, VECM	Unidirectional causality from RENW to CO_2_
Apergis and Payne (2014), 1980 − 2010	7 CA	FMOLS, Regime-Wise GRC	Bidirectional causality between RENW and CO_2_
Lin and Zhu (2017), 1970–2015	China	ARDL, VECM Granger causality	The influence of industrialization on energy and carbon intensity increases over time
Wang and Su (2019), 1990–2015	China	Granger causality	As the level of industrialization improves it helps growth and carbon emissions to be strongly decoupled in the long run
Mahmood et al. (2020), 1968–2014	Saudi Arabia	ARDL	Industrialization has inelastic but positive effect on the CO_2_ emissions
Wang et al. (2018), 1980–2014	China, India	Decoupling analysis, Impulse response	In both China and India, carbon emission intensity is the biggest contributor of decoupling, followed industrialization, among other variables

QARDL is Quantile Autoregressive Distributed Lag; ICT is Information and Communications Technology; FDV is financial development; CUP-FM is continuously updated fully modified; INSQTY is institutional quality; CUP-BC is continuously updated and bias corrected; GMM is dynamic system generalized method of moments; POLS is pooled ordinary least squares; FEM is fixed-effects model; SGMM is system generalized method of moments; GDP is gross domestic product; OLS is ordinary least squares; GLS is generalized least square; PMG is pooled mean group; CS-ARDL is cross-sectionally augmented autoregressive distributed lag; PSTR is panel smooth transition regression; MG is mean group; RENW is renewable energy; AMG is augmented mean group estimator; PCSE is panel corrected standard errors; DOLS is dynamic ordinary least squares; FMOLS is fully modified ordinary least squares; 2SLS is two-stage least squares; CCEMG is common correlated effects mean group; DCCE is dynamic common-correlated effects; VECM is vector error correction model; GRC is granger causality; CA is Central America; NREW is non-renewable energy; and OECD is the organization for economic cooperation and development; * is multiplicative sign. **Source**: Author's compilations

Despite empirical literature on environmental protection and quality, to the best of our knowledge, no studies have examined how ICT can distinctively complement renewable energy sources, institutional quality, and industrialization concurrently in mitigating CO_2_ emissions in BRICS countries using the novel CS-ARDL approach. It is on this basis that we embark on this study for the purpose of recommending policies that will help the economic bloc achieve faster and sustained environmental quality.

## Empirical methodology and data

### Empirical strategy

The preliminary empirical strategy used in this study include principal components approach/analysis (PCA), descriptive analysis, scatter plot (graph), panel unit root test (first-and second-generation), slope homogeneity test, cross-sectional dependence (CD) test, CIPS panel unit root tests, panel cointegration test (first- and second-generation), fully modified ordinary least square (FMOLS) and the dynamic OLS (DOLS), Dumitrescu and Hurlin (2012) panel causality, AMG and CCEMG estimation techniques.

This study employs a comprehensive econometric approach to analyze the data, following a systematic sequence of steps designed to ensure robustness and accuracy in the results. The methodology is outlined as follows: The analysis begins with Principal Component Analysis (PCA), which is used to reduce the dimensionality of the dataset while retaining the variability present in the data. This step helps in identifying the key components that explain the majority of the variance in the data. Following PCA, descriptive statistics are computed to provide a summary of the central tendency, dispersion, and shape of the dataset's distribution. This step offers a preliminary insight into the data's characteristics. Scatter plots are employed to visually examine the relationships between variables. This step aids in identifying any potential linear or nonlinear patterns, outliers, or clusters in the data. The slope homogeneity test is conducted to assess whether the slopes of the regression lines are homogeneous across different panels or groups in the dataset. This step is crucial for determining the appropriateness of pooling the data for further analysis.

The Cross-sectional Dependence (CD) test is performed to detect the presence of cross-sectional dependence in the panel data. This step is essential to ensure that the subsequent econometric techniques account for any interdependencies between cross-sectional units. The panel unit root test is applied to ascertain the stationarity properties of the variables. This step is necessary to avoid spurious regression results and to determine the appropriate level of differencing required for the analysis. Once the variables are confirmed to be stationary, a cointegration test is conducted to examine the long-run equilibrium relationships among the variables. This step helps in identifying any long-term associations that exist in the panel data. The panel causality test is employed to investigate the direction of causality between variables. This step provides insights into the dynamic interactions and causal relationships among the variables under study. Finally, the study employs panel Cross-sectionally Augmented Autoregressive Distributed Lag (CS-ARDL), Augmented Mean Group (AMG), and Common Correlated Effects Mean Group (CCEMG) estimations to analyze both the short-run and long-run relationships between the variables. The Cross-Sectional Augmented Autoregressive Distributed Lag (CS-ARDL) estimation method accounts for differences across countries in a panel data estimation (Chudik and Pesaran 2015). This approach is an extension of the ARDL model to panel data, and it is designed to handle heterogeneity across cross-sectional units (e.g., countries) by allowing for country-specific short-run dynamics and long-run relationships (Saba and Monkam 2024).

In the CS-ARDL model, the cross-sectional dependence among countries is addressed by augmenting the model with the cross-sectional averages of the dependent and independent variables (Chudik and Pesaran 2015). This approach helps to capture common factors that influence all countries in the panel, thereby controlling for cross-sectional dependence and heterogeneity (Chudik and Pesaran 2015). By incorporating country-specific effects and dynamics, the CS-ARDL model provides a more nuanced understanding of the relationships in the data, taking into account the unique characteristics and circumstances of each country in the panel (Chudik and Pesaran 2015).

**Fig. 2:**
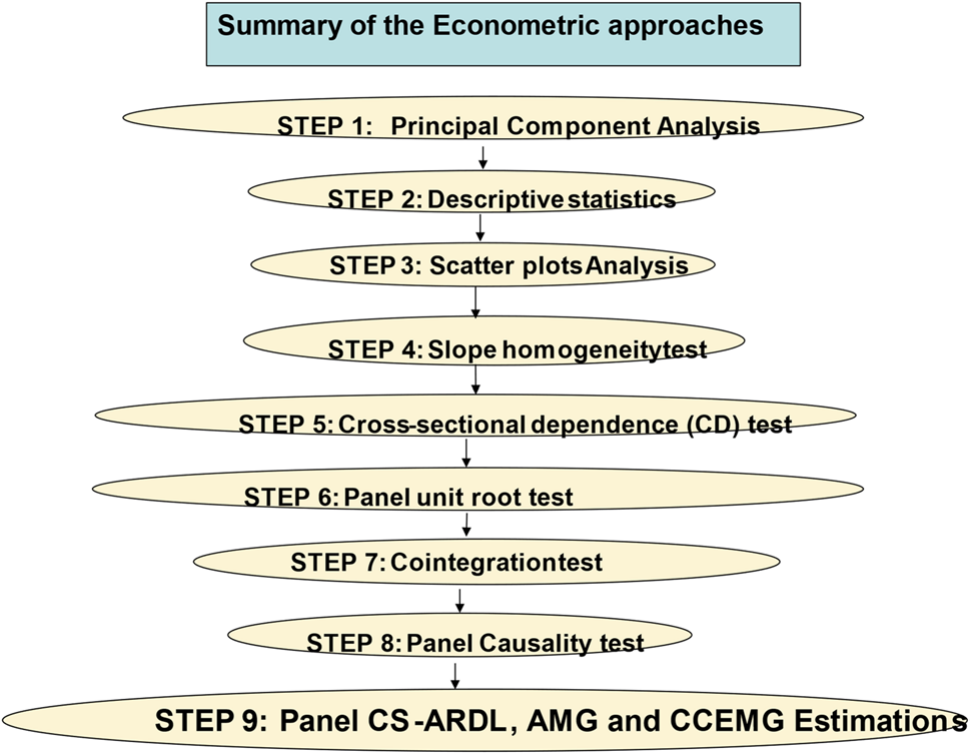
Summary of the Econometric approaches

These advanced econometric techniques account for cross-sectional dependence, heterogeneity, and dynamic interactions in the panel data. By following this methodological sequence, the study aims to provide a comprehensive and robust analysis of the relationships between the variables of interest, ensuring the validity and reliability of the econometric findings. To save space, we did not provide all the estimated equations for the aforementioned econometric techniques because they are readily available in other empirical literatures. But we rather focused our attention on the main estimation technique which is the CS-ARDL econometric technique. Figure 2 provides a visual representation of the methodological approach for the readers' ease of comprehension.

### Brief theoretical framework and empirical model specification

Theoretically, this paper utilizes the Cobb–Douglas production function (Cobb and Douglas 1928) with necessary modification for the purpose of investigating the relationship between ICT, renewable energy sources, industrialization, institutional quality and CO_2_ emissions within the context of BRICS economies. The aggregate growth function can be illustrated using a typical Cobb–Douglas production function with a constant rate of returns which is as follows:

1 $${Y}_{i,t}=f\left({K}_{i,t}, {L}_{i,t}\right)$$

where $Y, k and L$ is GDP, capital and effective labor, respectively. Theoretically, levels of income and CO_2_ emissions are related since it is widely believed that as economic activities take place in a country, it equally leads to the release of CO_2_ emissions in the economy (Kuznets 1955; Saba 2023a, 2023b). Moreover, rapid economic expansion is associated with a whole lot of factors (such as the use of ICT, energy, institutions etc.) during the production process, while increasing the proportion of renewable energy sources in total energy use such that it contributes to environmental sustainability by curbing CO_2_ emissions from dirty energy sources. Therefore, the augmented CO_2_ emission function can be expressed as:

2 $${LCO2EM}_{i,t}=f\left({X}_{i,t}\right)$$

3 $${LCO2EM}_{i,t}={\beta }_{0}+{\beth }_{0}{X}_{i,t}$$

4 $${LCO2EM}_{i,t}={\beta }_{0}+{\beth }_{0}{X}_{i,t}+ {\upepsilon }_{it}$$

where $LCO2EM$ and $X$ represents *log* of CO_2_ emissions (proxy for environmental sustainability) and regressors,^1^ respectively. The basic econometric models which were later transformed to CS-ARDL model and estimated can be found below:

- *Model 1*:where $\beta$, ${\beth }_{1},\dots ,{\beth }_{4},$ and ${\upepsilon }_{it}$ represents the constants, coefficient and the error term, respectively. Model 1 excludes the interaction terms between ICT and LIND, LICT and RESS, LICT and INSQTY, while the rest of the models (that is, model 5–8) does in a systemic manner one after the other.
  5 $${LCO2EM}_{i,t}= {\beta }_{1}+{\beth }_{1}{LGDPC}_{it}+{\beth }_{2}{LIND}_{it}+{\beth }_{3}{RESS}_{it}+{\beth }_{4}L{ICT}_{it}+{\beth }_{6}{INSQTY}_{it}+{\upepsilon }_{1it}$$

- Model 2: Capturing the interaction between LICT and LIND
  6 $${LCO2EM}_{i,t}= {\beta }_{1}+{\beth }_{1}{LGDPC}_{it}+{\beth }_{2}{LIND}_{it}++{\beth }_{3}{RESS}_{it}+{\beth }_{4}{LICT}_{it}+{\beth }_{5}{INSQTY}_{it} +{\beth }_{6}{ICT*LIND}_{it}+{\upepsilon }_{it}$$
- Model 3: Capturing the interaction between ICT and RESS
  7 $${LCO2EM}_{i,t}= {\beta }_{1}+{\beth }_{1}{LGDPC}_{it}+{\beth }_{2}{LIND}_{it}+{\beth }_{3}{RESS}_{it}+{\beth }_{4}{LICT}_{it}+{\beth }_{5}{INSQTY}_{it}+{\beth }_{6}{ICT*RESS}_{it}+{\upepsilon }_{it}$$
- Model 4: Capturing the interaction between ICT and INSQTY
  8 $${LCO2EM}_{i,t}= {\beta }_{1}+{\beth }_{1}{LGDPC}_{it}+{\beth }_{2}{LIND}_{it}+{\beth }_{3}{RESS}_{it}+ {\beth }_{4}{ICT}_{it}+{\beth }_{5}{INSQTY}_{it}+{\beth }_{6}{ICT*INSQ}_{it}+{\upepsilon }_{it}$$

We specify the CS-ARDL model below which took it bearing from the above equations:

9 $${\Delta LCO2EM}_{it}={\mathfrak{F}}_{i}+{\xi }_{i}\left({LCO2EM}_{i,t-1}-{\beth }_{i}{X}_{i,t-1}-{\beta }_{1i}{\overline{LCO2EM}}_{t-1}-{\beta }_{2i}{\overline{X}}_{t-1}\right)+\sum\nolimits_{j=0}^{p-1}{\gamma }_{ij}{\Delta LCO2EM}_{i,t-j}+\sum\nolimits_{j=0}^{v-1}{\Gamma }_{ij}{\Delta X}_{i,t-j}+{\varnothing }_{1i}\Delta {\overline{LCO2EM}}_{t}+{\varnothing }_{2i}\Delta {\overline{X}}_{t}+{u}_{it}$$

where $\Delta LCO2EM,$ ${X}_{i,t}$, ${\overline{LCO2EM}}_{t-1}$ & ${\overline{X}}_{t-1}$, ${\Delta LCO2EM}_{i,t-j} \& {\Delta X}_{i,t-j}$, $\Delta {\overline{LCO2EM}}_{t} \& \Delta {\overline{X}}_{t}$ and ${u}_{it}$ are dependent variable, all independent variables during the long run, mean of the dependent and explanatory variables in the long run, dependent and independent variables in the short run, mean dependent and independent variables during the short run and the error term, respectively. Furthermore, where j, t, ${\beth }_{1i}$, ${\gamma }_{1i}$, ${\Gamma }_{ij}$, ${\varnothing }_{1i}$ and ${\varnothing }_{2i}$ denotes cross-sectional dimension, time, coefficients of the independent variables, short-run coefficient of the dependent variable, short-run coefficients of the independent variables, mean of dependent variables and mean of independent variables in the short-run, respectively. The details of the dependent and independents variables can be found in Table 2. We used the AMG and CCEMG for robustness checks.

**Table 2:** Variable description and data sources

Variables	Description and definitions	Sources
Dependent variable		
Environmental Sustainability variable		
LCO2EM	Log of CO_2_ emissions (metric tons per capita): According to the WDI database, “*CO*_*2*_* emissions are those stemming from the burning of fossil fuels and the manufacture of cement. They include carbon dioxide produced during consumption of solid, liquid, and gas fuels and gas flaring*”	WDI database
Independent variables		
Renewable energy sources (RESS) variable was constructed using PCA for the below indicators		
RELO	Log of renewable electricity output (% of total electricity output). According to the WDI database, “*renewable electricity is the share of electricity generated by renewable power plants in total electricity generated by all types of plants*”	WDI database
REGC	Log of renewable energy consumption (% of total final energy consumption). According to the WDI database, “r*enewable energy consumption is the share of renewables energy in total final energy consumption*”	WDI database
Other explanatory variables		
LIND	Industrial, value added (% of GDP) (proxy for industrialization). According to the WDI database, “*it comprises value added in mining, manufacturing (also reported as a separate subgroup), construction, electricity, water, and gas. Value added is the net output of a sector after adding up all outputs and subtracting intermediate inputs. It is calculated without making deductions for depreciation of fabricated assets or depletion and degradation of natural resources*”	WDI database
LGDPC	GDP per capita (constant 2015 US$). According to the WDI database, “*GDP per capita is gross domestic product divided by midyear population. GDP is the sum of gross value added by all resident producers in the economy plus any product taxes and minus any subsidies not included in the value of the products. It is calculated without making deductions for depreciation of fabricated assets or for depletion and degradation of natural resources*”	WDI database
ICT variables was obtained from the indicators below via PCA		
LICT	ICT penetration is captured by a composite index of ICT indicators (which comprises of three indicators) by applying principal components method/analysis (PCA). These indicators include:	ITU database
	(i) mobile-cellular telephone subscriptions per 100 inhabitants (penetration of connected mobile lines) (mobT). According to the ITU database, “*Mobile cellular telephone subscriptions are subscriptions to a public mobile telephone service that provide access to the Public Switched Telephone Network (PSTN) using cellular technology. The indicator includes (and is split into) the number of postpaid subscriptions, and the number of active prepaid accounts (i.e. that have been used during the last three months). The indicator applies to all mobile cellular subscriptions that offer voice communications. It excludes subscriptions *via* data cards or USB modems, subscriptions to public mobile data services, private trunked mobile radio, telepoint, radio paging and telemetry services*”	
	(ii) fixed-telephone subscriptions per 100 inhabitants (penetration of connected fixed lines) (FLT). According to the ITU database, “*fixed telephone subscriptions refers to the sum of active number of analogue fixed telephone lines, voice-over-IP (VoIP) subscriptions, fixed wireless local loop (WLL) subscriptions, integrated services digital network (ISDN) voice-channel equivalents and fixed public payphones”*	
	(iii) percentage of Individuals using the Internet (percentage of population with access to the internet) (IAS). Internet users are individuals who have used the Internet (from any location) in the last 3 months. *According to the ITU database, “the Internet can be used *via* a computer, mobile phone, personal digital assistant, games machine, digital TV *etc.*”*	
Institutional quality (INSQTY) variable obtained from governance indicators		
WGIc	Control of Corruption: Estimate. According to the WGI database, *“control of corruption captures perceptions of the extent to which public power is exercised for private gain, including both petty and grand forms of corruption, as well as "capture" of the state by elites and private interests. Estimate gives the country's score on the aggregate indicator, in units of a standard normal distribution, i.e. ranging from approximately -2.5 to 2.5”*	WGI database
WGIp	Political stability and absence of violence/terrorism: Estimate. According to the WGI database, *“political stability and absence of violence/terrorism measures perceptions of the likelihood of political instability and/or politically-motivated violence, including terrorism. Estimate gives the country's score on the aggregate indicator, in units of a standard normal distribution, i.e. ranging from approximately -2.5 to 2.5”*	WGI database
WGIg	Government effectiveness: Estimate. According to the WGI database, *“government effectiveness captures perceptions of the quality of public services, the quality of the civil service and the degree of its independence from political pressures, the quality of policy formulation and implementation, and the credibility of the government's commitment to such policies. Estimate gives the country's score on the aggregate indicator, in units of a standard normal distribution, i.e. ranging from approximately -2.5 to 2.5”*	WGI database
WGIreg	Regulatory quality: Estimate. According to the WGI database, *“regulatory quality captures perceptions of the ability of the government to formulate and implement sound policies and regulations that permit and promote private sector development. Estimate gives the country's score on the aggregate indicator, in units of a standard normal distribution, i.e. ranging from approximately -2.5 to 2.5”*	WGI database
WGIr	Rule of law: Estimate. According to the WGI database, *“rule of law captures perceptions of the extent to which agents have confidence in and abide by the rules of society, and in particular the quality of contract enforcement, property rights, the police, and the courts, as well as the likelihood of crime and violence. Estimate gives the country's score on the aggregate indicator, in units of a standard normal distribution, i.e. ranging from approximately -2.5 to 2.5”*	WGI database
WGIv	Voice and accountability: Estimate. According to the WGI database, *“voice and accountability captures perceptions of the extent to which a country's citizens are able to participate in selecting their government, as well as freedom of expression, freedom of association, and a free media. Estimate gives the country's score on the aggregate indicator, in units of a standard normal distribution, i.e. ranging from approximately -2.5 to 2.5”*	WGI database

WDI represents World Bank's World Development Indicators. ITU represents International Telecommunication Union database. WGI represents World Bank's World Governance Indicators. There were missing data, but this was handled by means of interpolation and extrapolation of data.^2^**Source**: Author's compilations

### Data and variables description

This study utilized annual panel data for 5 BRICS countries (that is Brazil, China, India, Russia and South Africa) covering the period from 2000 to 2021. The data were sourced from three main databases, namely, the International Telecommunication Union (ITU), the World Bank's World Development Indicators (WDI), and the World Governance Indicators (WGI). The time span and countries used were selected based on data availability. The variables ICT, renewable energy sources, and institutional quality were obtained from the indicators listed in Tables 1 and 2 through the utilization of PCA. Table 2 list the variables used in this study. The justification for including the regressors in the model is explained briefly below:

i. *Renewable energy sources (RESS)*: Renewable energy has been identified in the environmental and energy literature as a potent tool that can help to combat environmental degradation and promote environmental sustainability, as the world grapples with the worsening impact of climate change across different sectors of the economy. Several studies that support this notion include Sharif et al. (2021), You and Kakinaka (2022), Saba and Ngepah (2022a, b), Saba and Biyase (2022), and Apergis et al. (2023), among others. Therefore, it is important to investigate the critical role that renewable energy has played in achieving environmental sustainability by the BRICS’s energy sector.
ii. *Industrial value added (proxy for industrialization) (INDU)*: Literature has shown that historically, industries have been a significant source of pollution due to their continuous demand for and use of fossil fuels in their production processes. The continuous use of energy pollutes the environment by increasing CO_2_ emissions, which indirectly affects economic growth (Anwar and Elfaki 2021; Elfaki et al. 2022). Therefore, it is important to investigate the role that the industrial sector plays in the path towards environmental sustainability in BRICS, especially when the variable is interacted with ICT. This is because the industrial sector utilizes ICT infrastructure in its production processes.
iii. *GDP per capita (proxy for levels of income/economic growth) (GDPC)*: The empirical literature has shown that the levels of income/output/economic growth contribute to environmental degradation at country-specific, regional, and global levels (see among others: Aslam et al. 2021; Saba 2023b; Boukhelkhal 2022). This is why the challenge of how to limit environmental pollution while preserving growth is a concern for policymakers today, and it is especially crucial for BRICS. Hence, further investigation into the role that economic growth plays using more advanced econometric approaches is crucial for providing policy directions.
iv. *Information and communications technology (ICT)*: Policymakers can leverage ICT diffusion to address critical policy concerns related to sustainable development goals, such as improving access to affordable and clean energy and mitigating global warming (climate action). This is because ICT is strongly associated with sustainable development as it helps reduce CO_2_ emissions by (a) lowering unnecessary transportation costs, (b) enhancing quality of life, (c) increasing productivity and financial affairs for households and firms, and (d) facilitating easy access to resources and knowledge, thereby reducing information asymmetry levels (Faisal et al. 2020; Chi and Meng 2022). Therefore, we are examining the role of ICT in promoting environmental sustainability in BRICS.
v. *Institutional quality (INSQ)*: In the extant literature, institutional quality has been strongly linked to environmental quality, as it influences every sector of the economy. The policies that political institutions adopt to provide the cultural and legal framework for the successful implementation of programs, projects, and societal activities could be tied to institutional quality (Yang et al. 2022; Jahanger et al. 2022). Therefore, examining its importance to environmental quality cannot be overemphasized, as it showcases the government's capacity and willingness to implement rules and laws meant to promote society in all its spheres (Islam et al. 2021; Haldar and Sethi 2021). Environmental policies cannot operate outside the established institutions in a society.

## Empirical results and discussion

### Preliminary analysis

#### Principal component analysis

**Fig. 3:**
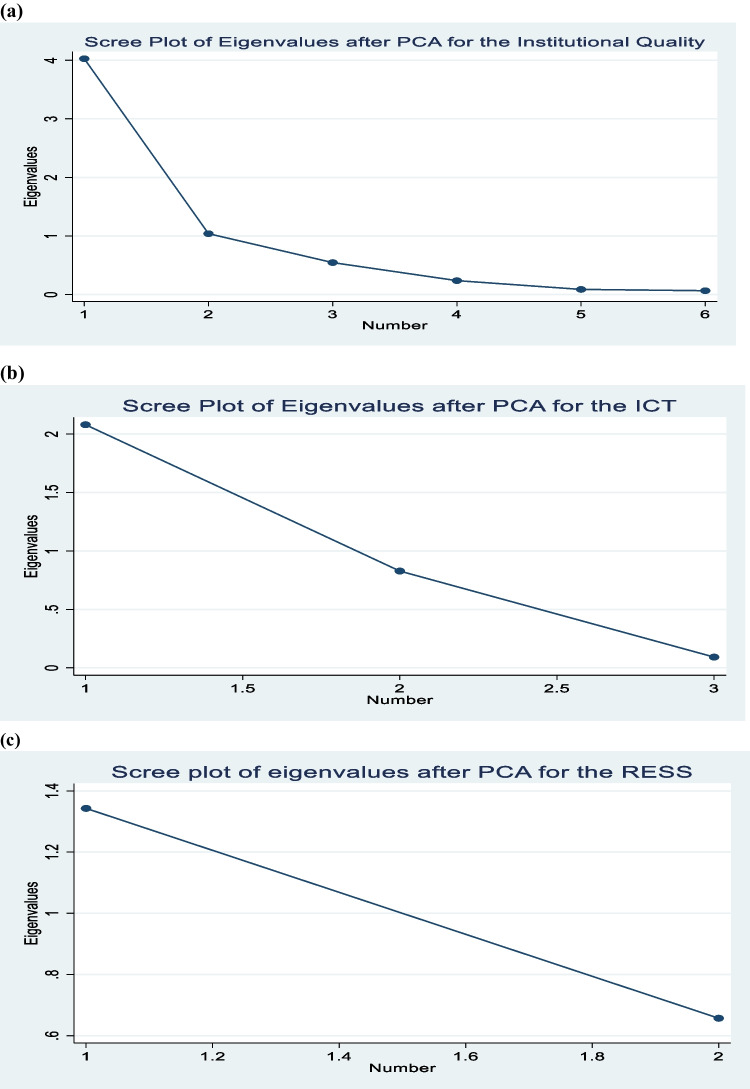
**(a)** Scree plot for institutional quality; (**b**) Scree plot for ICT; and (**c**) Scree plot for renewable energy

Table 3 presents the principal component approach and correlation matrix results for institutional quality (INSQTY), LICT and renewable energy sources (RESS) variables. We first started by testing whether or not there are some degree of association between the indicators used to generate an index for each of the variables, that is, INSQTY, LICT and RESS. The results in Panel A, B and C show that the indicators are strongly correlated, hence, we proceeded to the estimation of the PCA given that the condition of the indicators being correlated was filled (Saba and Ngepah 2022a, 2022b, 2022c). To create a composite index for institutional quality, we selected the first principal component that explains the highest percentage of the total variation. We followed the same approach for LICT and RESS. We selected the first component for the INSQTY, LICT, and RESS variables because its eigenvalue accounts for the highest percentage of the total variation, which is 4.03%, 2.08%, and 1.34%, respectively. The scree plots in Fig. 3 further supports our results.

**Table 3:** Principal component and correlation matrix results for institutional quality, ICT and renewable energy sources variables

Panel (A): Institution quality variables							
Principal component results							
Compnnt	Eigenvalue	Difference	Proportion	Cumulative			
Compnnt 1	4.028	2.989	0.671	0.671			
Compnnt 2	1.039	0.494	0.173	0.845			
Compnnt 3	0.545	0.308	0.091	0.935			
Compnnt 4	0.237	0.150	0.040	0.975			
Compnnt 5	0.087	0.022	0.015	0.989			
Compnnt 6	0.065		0.011	1.000			
Principal components eigenvectors results							
Variables	Compnnt 1	Compnnt 2	Compnnt 3	Compnnt 4	Compnnt 5	Compnnt 6	Unexplained
WGIv	0.330	0.651	0.429	-0.112	0.176	0.490	0.1215
WGIr	0.412	0.417	-0.335	0.465	0.241	-0.519	0.1348
WGIreg	0.447	-0.187	0.274	-0.654	0.142	-0.492	0.1595
WGIp	0.351	-0.551	0.458	0.539	0.235	0.133	0.1889
WGIg	0.409	-0.253	-0.646	-0.216	0.281	0.475	0.260
WGIc	0.481	-0.014	-0.046	0.076	-0.870	0.065	0.0685
Correlation matrix results i		ii	iii	iv	v	vi	
(i) WGIv	1.000						
(ii) WGIr	0.727*** (0.000)	1.000					
(iii) WGIreg	0.536*** (0.000)	0.558*** (0.000)	1.000				
(iv) WGIp	0.194*** (0.000)	0.320*** (0.000)	0.722*** (0.000)	1.000			
(v) WGIg	0.247*** (0.000)	0.654*** (0.000)	0.711*** (0.000)	0.544*** (0.000)	1.000		
(vi) WGIc	0.606*** (0.000)	0.789*** (0.000)	0.837*** (0.000)	0.668*** (0.000)	0.789*** (0.000)	1.000	
Panel (B): ICT variables							
Principal component results							
Component	Eigenvalue	Difference	Proportion	Cumulative			
Compnnt 1	2.080	1.252	0.694	0.694			
Compnnt 2	0.828	0.736	0.276	0.969			
Compnnt 3	0.092		0.031	1.000			
Principal components eigenvectors results							
Variable	Compnnt 1	Compnnt 2	Compnnt 3	Unexplained			
Fixed-telephone	0.377	0.922	0.087	0.705			
Mobile-telephone	0.646	-0.329	0.689	0.133			
Internet access	0.664	-0.204	-0.719	0.082			
Correlation matrix results							
Variables	i	ii	iii				
(i) Fixed-telephone	1.000						
(ii) Mobile-telephone	0.260*** (0.010)	1.000					
(iii) Internet access	0.359*** (0.000)	0.902*** (0.000)	1.000				
Panel (C): Renewable energy sources variables							
Principal component results							
Component	Eigenvalue	Difference	Proportion	Cumulative			
Compnnt 1	1.343	0.686	0.672	0.672			
Compnnt 2	0.657		0.329	1.000			
Principal components eigenvectors results							
Variable	Compnnt 1	Compnnt 2	Unexplained				
RELO	0.707	0.707	0.329				
REGC	0.707	-0.707	0.329				
Correlation matrix results							
Variables	i	ii					
(i) RELO	1.000						
(ii) REGC	0.343*** (0.000)	1.000					

^***^p < 0.01; **p < 0.05; *p < 0.1, p-value in parentheses. Where compnnt is component **Source:** Author’s computation using WDI, WGI and ITU data

#### Summary statistics and scatter plot analysis

Table 4 (Panel A) presents the summary statistics results for the variables. We observed that for the series, the mean (or median) value of LCO2EM, LIND, LGDPC, LICT, RESS and INSQTY is around 1.419 (1.846), 3.389 (3.355), 8.568 (8.900), 0.100 (0.474), -0.023 (0.032) and 2.20E-09 (-0.254), respectively. The maximum and minimum values of the variables are found to be approximately between 9.392 and -5.045, respectively. 0.841, 0.240, 0.814, 1.361, 1.164 and 2.007 for LCO2EM, LIND, LGDPC, LICT, RESS and INSQTY, respectively, which indicate the variation in series. The series with the negative and positive values of skewness shows a negatively and positively skewed distribution for the variables, respectively.

**Table 4:** Discriptive Statistics results

Statistics	LCO2EM	LIND	LGDPC	LICT	RESS	INSQTY
Mean	1.419	3.389	8.568	0.100	-0.023	2.20E-09
Median	1.846	3.355	8.900	0.474	0.032	-0.254
Maximum	2.454	3.862	9.392	1.719	1.715	3.873
Minimum	-0.117	2.901	6.717	-5.045	-1.976	-3.982
Std. Dev	0.841	0.240	0.814	1.361	1.164	2.007
Skewness	-0.337	0.465	-0.931	-1.215	0.016	0.260
Kurtosis	1.564	2.761	2.458	4.566	1.670	2.215
Jarque–Bera	9.542	3.498	14.272	31.678	6.710	3.366
Probability	0.008	0.172	0.001	0.000	0.035	0.186
Observations	91	91	91	91	91	91

**Source**: Author's computations

The summary statistics presented in Table 4 (Panel A) provide insights into the distribution of the variables in the study, which include CO_2_ emissions (LCO2EM), industrialization (LIND), GDP per capita (LGDPC), information and communications technology (ICT), renewable energy sources (RESS), and institutional quality (INSQTY). The mean (average) and median (middle value) of the variables provide a sense of the central tendency. For example, the mean value of CO_2_ emissions is 1.419, and the median is 1.846, indicating that the distribution is skewed since the mean is less than the median. The range of values, indicated by the maximum and minimum, shows the extent of variation in the data. For instance, CO_2_ emissions vary between -5.045 and 9.392, suggesting a wide range of emissions levels across the observations. The standard deviation values (0.841 for LCO2EM, 0.240 for LIND, etc.) measure the dispersion of the data around the mean. A higher standard deviation indicates greater variability. For example, ICT has a relatively high standard deviation of 1.361, suggesting diverse levels of ICT adoption among the observations. The skewness values indicate the asymmetry of the distribution. A positive skewness (e.g., 0.465 for LIND) means the tail on the right side of the distribution is longer, while a negative skewness (e.g., -0.337 for LCO2EM) indicates a longer tail on the left side. This information helps understand the distribution's shape and potential outliers. The kurtosis values measure the 'tailedness' of the distribution. A kurtosis greater than 3 (e.g., 4.566 for ICT) indicates a distribution with heavier tails and a sharper peak compared to a normal distribution, suggesting a higher likelihood of extreme values. The variability in CO_2_ emissions, GDP per capita, and ICT suggests differing levels of economic development and technological adoption among the countries in the sample.

Based on the summary statistics provided, we can make some descriptive observations about the levels of environmental sustainability, ICT, renewable energy, industrialization, and institutional quality in the dataset: *Firstly*, the mean and median values of CO_2_ emissions (LCO2EM) suggest that there is a wide range of emissions levels across the observations, with some countries having significantly higher emissions than others. The negative skewness indicates that the distribution is skewed towards lower emissions, but the presence of high kurtosis suggests that there are also countries with extremely high emissions levels. *Secondly*, the mean and median values for ICT are relatively low, indicating that overall ICT adoption may be limited in the sample. However, the high standard deviation and kurtosis suggest that there is significant variability, with some countries having much higher levels of ICT adoption.

*Thirdly*, the mean value of renewable energy sources is slightly negative, while the median is positive, indicating a skewed distribution with a majority of countries having low levels of renewable energy usage. However, the presence of countries with higher usage is also indicated by the positive skewness and kurtosis. *Fourthly*, the mean and median values for industrialization are relatively close, suggesting a more symmetric distribution. However, the positive skewness and high kurtosis indicate that there are countries with significantly higher levels of industrialization, contributing to the tail on the right side of the distribution. *Fifthly*, the mean value is very close to zero, and the median is negative, suggesting that overall institutional quality may be low in the sample. The positive skewness and kurtosis indicate that while most countries have lower institutional quality, there are a few countries with much higher quality.

Overall, the levels of environmental sustainability, ICT, renewable energy, industrialization, and institutional quality vary widely across the countries in the sample. There are indications of both low and high levels of each variable, with significant variability and skewness in the distributions. These descriptive insights serve as a foundation for deeper analysis and the application of the various econometric techniques utilized in this study to elucidate the relationships among these variables and their effects on environmental sustainability.

**Fig. 4:**
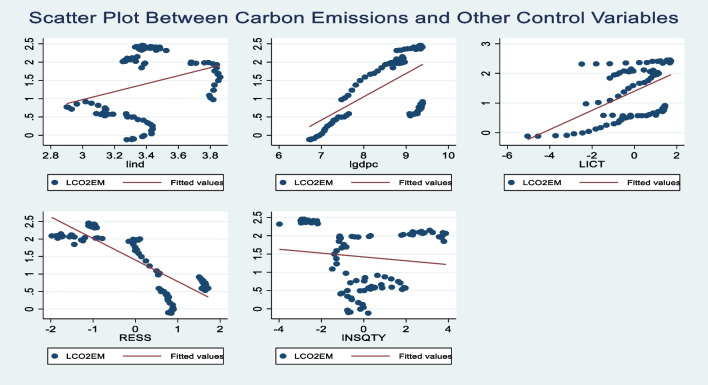
Scatter plots revealing the relationship between CO2 emissions and the regressors

Figure 4 presents scatter plots that visually demonstrate the relationship between CO_2_ emissions and the explanatory variables. The scatter plot graphs reveal that CO_2_ emissions has a positive relationship with industrialization, GDPC and ICT, while it has a negative relationship with renewable energy sources and institutional quality. Therefore, this initial relationship was provided only to give an indication of the potential relationship that may exist between CO_2_ emissions and the other explanatory variables in BRICS within the time periods examined. It should be noted that the positive relationship between CO_2_ emissions and some of the explanatory variables cannot be properly validated since the scatter plots graphical approach does not account for cross-sectional dependence issues.

#### *Slope homogeneity, cross-sectional dependence (CD)*, *panel unit root, and cointegration analysis*

To begin our study, we conducted a slope homogeneity test suggested by Pesaran and Yamagata (2008), and the results of the test can be found in Table 5. Here both test statistics (delt and adjusted delta) and probability values seem to reject the assumption of homogenous slope coefficients at the 1% significance level. This would suggest the presence of strong country heterogeneity (in the long run) for the variables under consideration. To account for cross-sectional dependence, we tested for cross-sectional dependence in the data using Pesaran (2004) and Breusch and Pagan (1980) LM test techniques. The two approaches result in Table 6 show that there is evidence of cross-sectional dependence in the series, with *p-*values for the statistic being statistically significant at 1%. Therefore, the null hypothesis (cross-sectional independence) is rejected. This implies that levels of all the variables in one member of BRICS depend on the movements of these aspects in at least one other member within the BRICS.

**Table 5:** Slope homogeneity results

Test statistics (Delta)	Value	p-value
${\Delta }_{delt}$	6.253***	0.000
${\Delta }_{adj delt}$	7.970***	0.000

^***^, ** and * denote significance at 1%, 5% and 10%, respectively. **Source:** Author’s Computations

**Table 6:** Cross-sectional dependence (CD) test results

	Pesaran test		Breusch-Pagan LM test	
Variables	Statistic	P-value	Statistic	P-value
LCO2EM	9.08***	0.000	31.215***	0.000
LIND	5.25***	0.000	25.339***	0.000
LGDPC	12.80***	0.000	48.083***	0.000
LICT	13.28***	0.000	46.862***	0.000
RESS	1.41***	0.000	23.305***	0.000
INSQTY	-2.30***	0.021	30.772***	0.000

^***^ p < 0.01, ** p < 0.05, and * p < 0.1 are significance level respectively at denote rejection of null hypothesis. **Source:** Author’s computations

Although the asymptotic results of all first-generation panel unit root (that is, Levin-Lin-Chu (LLC) (Levin et al. 2002) and Im-Pesaran-Shin (IPS) (Im et al. 2003)) tests rely on the assumption of cross-sectional independence, we still tested the first-generation panel unit root. However, their reliability was less important. Table 7 shows the results at levels and first differences to determine whether the variables were integrated of order zero or one. The stationarity tests for the two techniques had a null hypothesis of a unit root. While some variables were not stationary at levels, a critical examination of the results in the last column of Table 7 shows that all variables had an integrated order of 1 at a significance level of at least 1%, indicating perfect stationarity at first differences (except for two variables). Like the first-generation panel unit root results, the second-generation panel unit root (CIPS) results of Pesaran (2007) showed that the series were integrated of order 1 at least at a 1% significance level (see Table 8). This indicates that we can proceed to test the long-run equilibrium relationship between the series using the second-generation cointegration approach. More importantly, based on these estimates and the aforementioned unit root and cross-dependency tests, it does confirm the adequacy of the use of the panel CS-ARDL estimator to determine the existence of a possible relationship between all the variables used in this study.

**Table 7:** Panel unit root test results

Series	Model	Levels	First Difference
LCO2EM	LLC	-2.683*** (0.004)	-1.447* (0.074)
	IPS	-1.094 (0.137)	-1.726** (0.042)
LIND	LLC	0.176 (0.570)	-1.859** (0.032)
	IPS	0.283 (0.612)	-2.213*** (0.014)
LGDPC	LLC	-2.589*** (0.005)	-2.956*** (0.002)
	IPS	-0.539 (0.295)	-1.723** (0.043)
LICT	LLC	-5.278*** (0.000)	-4.839*** (0.000)
	IPS	-4.440*** (0.000)	-2.370*** (0.008)
RESS	LLC	-1.529 (0.263)	-2.544*** (0.006)
	IPS	-0.238 (0.406)	-3.098*** (0.001)
INSQTY	LLC	1.633 (0.949)	-2.267*** (0.012)
	IPS	1.696 (0.955)	-1.987** (0.023)

Null: Unit root (assumes common unit root process): Levin, Lin & Chu (t*). Null: Unit root (assumes individual unit root process): Im, Pesaran and Shin (W-stat). *** p < 0.01, ** p < 0.05, and * p < 0.1 are significance level respectively. **Source:** Author's computations

**Table 8:** CIPS Panel unit root test results

Variables	Levels	1st Difference
LCO2EM	-1.711	-2.480***
LIND	-2.402**	-3.123***
LGDPC	-1.649	-2.726***
LICT	-1.003	-3.357***
RESS	-2.047	-4.436***
INSQTY	-2.160	-3.633***

^*^, ** and *** denote statistically significant at the 1%, 5%, and 10% level respectively. The critical values of CIPS test at 10%, 5% and 1% significance levels are: -2.21, -2.34 and -2.6 for no intercept nor trend, respectively. **Source:** Author's computations

To investigate the long-run equilibrium relationship between the variables, we used the Pedroni and Westerlund panel cointegration tests of cointegration. The results of the Pedroni cointegration test (refer to Table 9) reveal that there exists a long-run equilibrium relationship between the variables since the values of the *rho*-statistics, *PP*-statistics, and *ADF*-statistics were statistically significant at least at the 10% significance level for the Within-dimension estimates. While for the Between-dimension estimates the values of the *PP*-statistics, and *ADF*-statistics were statistically significant at least at the 10% significance level which implies that there is long-run equilibrium relationship between the variables. To perform robustness checks within the context of cointegration test, and account for the presence of cross-sectional dependence in our data, we employed the Westerlund (2007) cointegration test. The results of the test (refer to Table 10) reveal that the values of ${G}_{t}$, ${G}_{a}$, ${P}_{t}$ and ${P}_{a}$ statistics were statistically significant at least at the 10% significance level which implies that there is also long-run equilibrium relationship between the variables despite the presence of cross-sectional dependence in the data.

**Table 9:** Pedroni tests for cointegration

Tests	Within-dimension (panel estimates)		Between-dimension (group estimates)	
	Statistic	p-value	Statistic	p-value
$Panel vv-{\text{statistic}}$	-1.284	0.901		
$Panel rho-statistics$	1.038**	0.050	2.414	0.992
$Panel PP-statistics$	-3.801***	0.000	-1.363*	0.086
$Panel ADF-statistics$	-5.316***	0.000	-3.849***	0.000

^***^, ** and * denote significance at 1%, 5% and 10%, respectively. **Source:** Author’s Computations

**Table 10:** Westerlund panel cointegration tests

Statistic	Value	Z-value	P-value	Robust P-value
${G}_{t}$	-0.858**	4.196	0.000	0.051
${G}_{a}$	-1.026*	3.784	0.000	0.090
${P}_{t}$	-1.918**	3.135	0.099	0.050
${P}_{a}$	-1.026*	2.761	0.997	0.091

^*^, ** and *** represent significance at the 1%, 5%, and 10% levels respectively; number of replications to obtain bootstrapped p-values is set to 100; bandwidth is selected according to the data depending rule $4{(\frac{T}{100})}^{2/9}\approx 3$ recommended by Newey and West (1994); Barlett is used as the spectral estimation method. **Source:** Author’s Computations

We used FMOLS and DOLS techniques proposed by Pedroni (2001, 2004) to estimate the long-run coefficients of the explanatory variables, as these techniques address serial correlation and endogeneity issues better than OLS. The R-squared and Adjusted R-squared values, which were above 80% for both approaches, demonstrate that our models were correctly specified. Therefore, we can proceed to interpret our estimated results. For Column 1, in the long run, renewable energy sources and institutional quality (except for DOLS) significantly contribute to reducing CO_2_ emissions, while ICT, industrialization, and GDP per capita have the opposite effect. These results are consistent with the findings of Adebayo (2020), Teng et al. (2021), Adebayo and Kalmaz (2021), Azam et al. (2021a, b, 2022), Raihan and Tuspekova (2022a, b, c, d), among others. This underscores the importance of exploring the role that ICT could play when it is interacted with the other explanatory variables. When LICT is interacted with industrialization (refer to Column 2 of Table 11), the results reveal that both variables reduce CO_2_ emissions. However, for the impact of the interaction between LICT and renewable energy on CO_2_ emissions, the opposite is observed in the long-run cointegration (refer to Column 3 of Table 11). While the impact of the interaction between LICT and institutional quality on CO_2_ emissions also reduces CO_2_ emissions (refer to Column 4 of Table 11). This leads us to investigate the causality relationships that may exist between our variables of interest.

**Table 11:** FMOLS and DOLS estimates

	(1)		(2)		(3)		(4)	
Variables	FMOLS	DOLS	FMOLS	DOLS	FMOLS	DOLS	FMOLS	DOLS
LIND	0.371***	0.075	0.746***	0.370***	0.330***	0.124	0.262***	0.018
	(0.000)	(0.786)	(0.000)	(0.015)	(0.000)	(0.245)	(0.000)	(0.871)
LGDPC	0.569***	0.133***	0.759***	0.518***	0.609***	0.120***	0.042***	0.160***
	(0.000)	(0.000)	(0.000)	(0.006)	(0.000)	(0.004)	(0.000)	(0.000)
LICT	0.018*	0.187***	0.715***	-0.704***	-0.344***	0.119***	0.254***	0.115***
	(0.089)	(0.000)	(0.000)	(0.000)	(0.000)	(0.000)	(0.000)	(0.002)
RESS	-0.142**	-0.554***	-0.214***	-0.158***	-0.103***	-0.560***	-0.528***	-0.544***
	(0.000)	(0.007)	(0.000)	(0.000)	(0.006)	(0.000)	(0.000)	(0.000)
INSQTY	-0.020***	-0.067***	0.357***	-0.013***	-0.198***	-0.091***	-0.057***	-0.074***
	(0.001)	(0.002)	(0.000)	(0.429)	(0.007)	(0.000)	(0.000)	(0.000)
LIND*LICT			-0.304***	-0.204***				
			(0.000)	(0.001)				
RESS*LICT					0.229***	0.082***		
					(0.000)	(0.000)		
INSQTY*LICT							-0.032***	0.010
							(0.000)	(0.464)
R^*2*^	0.99	0.93	0.84	0.99	0.89	0.93	0.92	0.91
Adj R^*2*^	0.99	0.94	0.88	0.99	0.87	0.92	0.92	0.91
Obs	82	82	82	82	82	82	82	82

^***^ p < 0.01, ** p < 0.05, * p < 0.1. Probability values in bracket, while th dependent variable is CO_2_ emissions. **Source:** Author’s Computations

### Panel causality and CS-ARDL, AMG and CCEMG estimates analysis

This section analysed the causal relationship between the series under review. Table 12 presents the panel causality test results. In Table 12, two-way causality exists between GDP per capita and CO_2_ emissions. This implies that the two variables depend on each other, and it is consistent with Esso and Keho (2016) and Boukhelkhal’s (2022) findings but contradicts Acheampong's (2018) study. While unidirectional causality runs from: CO_2_ emissions to industrialization which is in line with the findings of Shahbaz et al. (2014); ICT to CO_2_ emissions which is consistent with the findings of Faisal et al. (2020); and renewable energy sources to CO_2_ emissions.

**Table 12:** Dumitrescu and Hurlin (2012) panel causality test results

Model	Null hypothesis	W-statistic	Zbar-statistic	p-value	Direction of relationship observed	Conclusion
1	LIND $\nrightarrow$ LCO2EM	2.155	1.216	0.224	LCO2EM → LIND	Unidirectional causality
	LCO2E $\nrightarrow$ LIND	7.084**	7.203	0.000		
2	LGDPC $\nrightarrow$ LCO2EM	2.763**	1.955	0.051	LGDPC ↔ LCO2EM	Bidirectional causality
	LCO2E $\nrightarrow$ LGDPC	8.711***	9.180	0.000		
3	LICT $\nrightarrow$ LCO2EM	8.466***	8.882	0.000	LICT → LCO2EM	Unidirectional causality
	LCO2EM $\nrightarrow$ LICT	0.568	-0.712	0.476		
4	RESS $\nrightarrow$ LCO2EM	6.232***	6.168	0.000	RESS → LCO2EM	Unidirectional causality
	LCO2EM $\nrightarrow$ RESS	1.967	0.988	0.323		
5	INSQTY $\nrightarrow$ LCO2EM	1.051	-0.136	0.892	INSQTY $\nrightarrow$ LCO2EM	No causality
	LCO2EM $\nrightarrow$ INSQTY	2.485	1.577	0.115		

↔ and → denote bidirectional and unidirectional causality respectively. $\nrightarrow$ denote does not homogeneously cause (i.e.**H**_**0**_). *** p < 0.01, ** p < 0.05, * p < 0.1. **Source:** Author’s Computations

One of the reasons that could be responsible for this is that renewable energy sources like wind or solar power may be located in remote areas in the BRICS countries where the electricity grid infrastructure is not well-developed. In these cases, the energy generated from renewable sources may need to be transported long distances, which can result in some carbon emissions from the transportation of equipment, materials, and personnel. There was no causality between institutional quality and CO_2_ emissions which implies that they are independent of each other in BRICS economy. Even if institutions exist to regulate carbon emissions, they may not be enforced effectively. This could be due to corruption, lack of resources, or other factors that limit the ability of institutions to implement and enforce regulations. We rejected/accepted the null hypothesis that there was no causation for each Chi square-value statistic since their p-values were less/greater than the 10% significance level.

The causal relationships observed in the panel causality test results can be justified using various economic and environmental theories: *firstly*, the two-way causality between GDP per capita and CO_2_ emissions can be explained by the Environmental Kuznets Curve (EKC) theory. The EKC hypothesis suggests that environmental degradation initially increases with economic growth, reaches a peak, and then declines as the economy continues to grow (Grossman and Krueger 1991). This theory supports the bidirectional causality found in this study and aligns with the findings of Esso and Keho (2016) and Boukhelkhal (2022) as mentioned earlier. *Secondly*, the unidirectional causality from CO_2_ emissions to industrialization can be explained by the Pollution Haven Hypothesis (PHH). According to the PHH, countries with less stringent environmental regulations attract more pollution-intensive industries, leading to higher CO_2_ emissions (Copeland and Taylor 2017). This causal relationship is consistent with the findings of Shahbaz et al. (2014) as mentioned earlier.

**Fig. 5:**
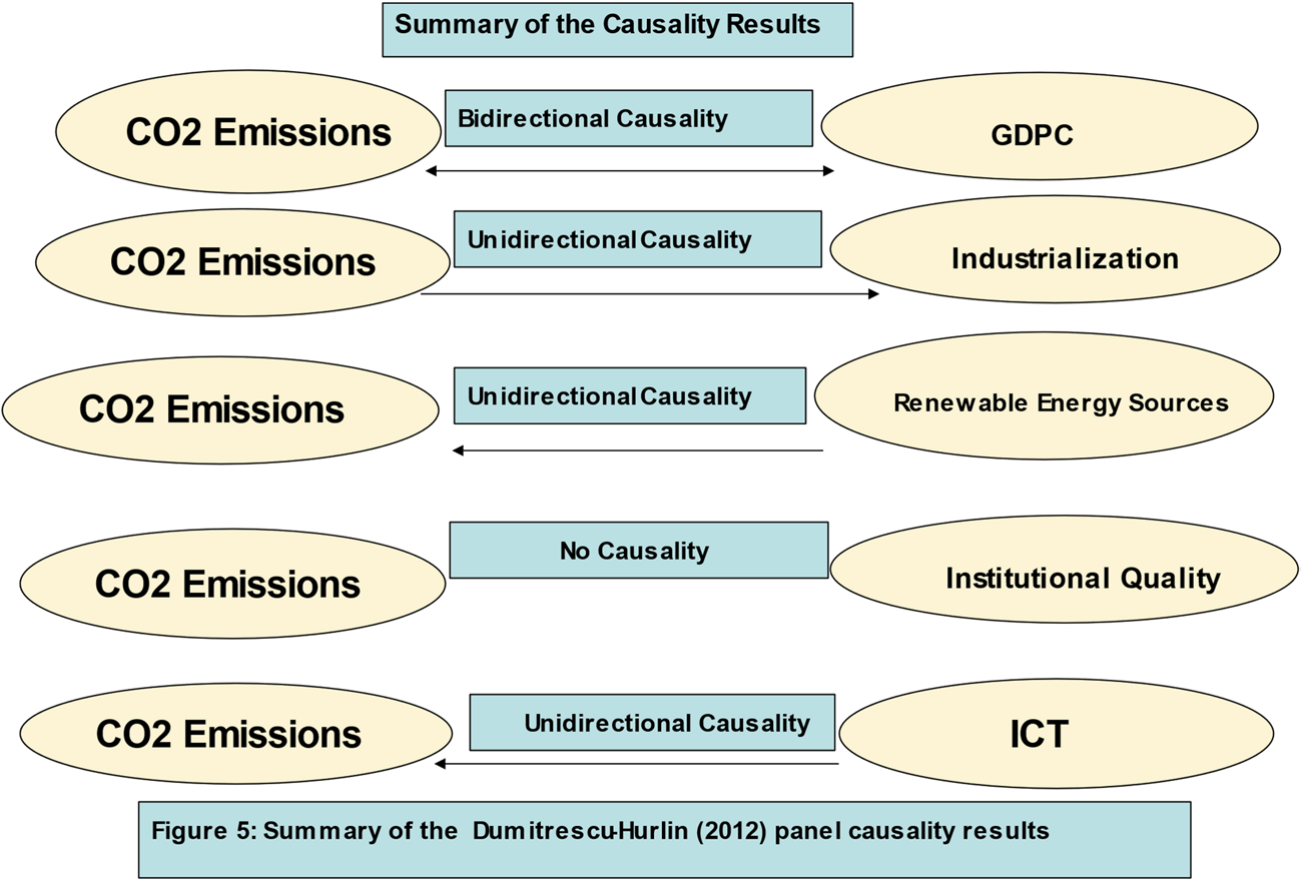
Dumitrescu and Hurlin (2012) panel causality test results

*Thirdly*, the causality from ICT to CO_2_ emissions can be explained by the theory of ecological modernization. This theory suggests that technological advancements, such as ICT, can lead to more efficient and less resource-intensive production processes, thereby reducing environmental impacts (Mol and Spaargaren 2000). This is consistent with the findings of Faisal et al. (2020) as mentioned earlier. *Lastly*, the unidirectional causality from renewable energy sources to CO_2_ emissions is supported by the theory of sustainable development. Renewable energy sources, such as solar and wind, are cleaner and produce fewer emissions compared to fossil fuels. The adoption of renewable energy can lead to a reduction in CO_2_ emissions, supporting the transition to a more sustainable energy system (Holden et al. 2014). Figure 5 presents the visual summary of the Dumitrescu and Hurlin (2012) panel causality test results.

Table 13 presents the panel CS-ARDL results. Beginning with the estimates of the Error Correction Term (ECT) for all regression models, the values are -1.344 (refer to Column 1), -1.413 (refer to Column 2), -1.061 (refer to Column 3), and -1.390 (refer to Column 4), which are significant at least at the 1% level of significance. These values suggest that there is a strong negative relationship between the deviations from the long-run equilibrium and the short-run changes in the CO_2_ emissions variable. Specifically, the negative ECT values indicate that any deviations from the long-run equilibrium will be corrected at a rate of approximately 1.344, 1.413, 1.061, and 1.390 units per period, respectively, suggesting that the CO_2_ emissions variable will adjust back towards its equilibrium level relatively quickly. Additionally, the R-squared values for all the models are above 50%, which implies that our models are correctly specified.

**Table 13:** Panel CS-ARDL estimates

	(1)	(2)	(3)	(4)
Variables	CS-ARDL 1	CS-ARDL 2	CS-ARDL 3	CS-ARDL 4
Short Run Est				
ΔLIND	0.430*	1.318*	0.253	0.673**
	(0.234)	(0.770)	(0.279)	(0.352)
ΔLGDPC	0.449**	2.276**	0.740**	1.054***
	(0.180)	(0.951)	(0.382)	(0.235)
ΔLICT	0.071	-0.093	0.077	-0.151**
	(0.084)	(0.241)	(0.049)	(0.072)
ΔRESS	-0.361**	-0.351**	-0.652***	-0.292**
	(0.148)	(0.158)	(0.266)	(0.152)
ΔINSQTY	-0.006	0.029	-0.042*	-0.041
	(0.028)	(0.037)	(0.023)	(0.033)
ΔLIND*LICT		-0.094*		
		(0.053)		
ΔRESS*LICT			0.154*	
			(0.084)	
ΔINSQTY*LICT				-0.022*
				(0.012)
Adjust. Term				
ECT	-1.344***	-1.413***	-1.061***	-1.390***
	(0.114)	(0.258)	(0.114)	(0.197)
Long Run Est				
lr_lind	0.301*	1.355*	0.220	0.397*
	(0.176)	(0.819)	(0.266)	(0.223)
LR_LGDPC	0.329**	2.072**	0.645**	0.720***
	(0.139)	(1.009)	(0.321)	(0.107)
LR_LICT	0.054	-0.071	0.075	-0.118
	(0.065)	(0.225)	(0.053)	(0.072)
LR_RESS	-0.270**	-0.309**	-0.650**	-0.206
	(0.117)	(0.141)	(0.291)	(0.131)
LR_INSQTY	-0.006	0.009	-0.040*	-0.025
	(0.023)	(0.028)	(0.018)	(0.018)
LR_IND*ICT		-0.075		
		(0.048)		
LR_RESS*LICT			0.148*	
			(0.083)	
LR_INSQTY*LICT				-0.018
				(0.012)
Obs	81	81	85	81
R-squared	0.85	0.72	0.99	0.69

Standard errors in parentheses; *, ** and *** represent significance at the 1%, 5%, and 10% levels respectively. **Source:** Author’s Computations

For the first model which shows the results without the interaction term variables, in Column 1 of Table 13, the CS-ARDL results show that both in the short and long run, at a 10% level of significance, the impact of industrialization and GDP per capita on CO_2_ emissions is significant and positive. Specifically in the short run, a 1% increase in industrialization and GDP per capita led to a 0.43% and 0.45% increase in CO_2_ emissions, respectively. These findings are consistent with the studies of Ike et al. (2020), Aslam et al. (2021) and Zafar et al. (2022). In the short and long run, it is only renewable energy sources that has a significant and negative impact on CO_2_ emissions at least at 5% level of significance. In the short and long run, a 1% increase in renewable energy sources leads -0.36% and -0.27% decrease in CO_2_ emissions, respectively. This implies that both in the short and long run renewable energy sources promotes environmental sustainability in BRICS countries. Both the ICT and institutional quality variables in the short and long run does not significantly impact CO_2_ emissions. This contradicts the findings of Lu (2018) and Haseeb et al. (2019) Warsame et al. (2022) because they used different methodologies and focused on different regions.

For the second model, which shows the impact of the interaction between industrialization and ICT on CO_2_ emissions reveal that in Column 2 of Table 13, the CS-ARDL results show that in the short run the interaction between industrialization and ICT has a negative and significant impact on CO_2_ emissions. This indicate that a 1% increase in the interaction between industrialization and ICT leads to -0.09% decrease in CO_2_ emission, while in the long run the interaction between the two variables does not have a significant impact on CO_2_ emission. This suggests that industrialization supported by ICT can help to reduce CO_2_ emissions in BRICS in the short run. This concurs with Asumadu-Sarkodie and Owusu (2017) and Elfaki, et al.’s (2022) studies and counters with the study of Asumadu-Sarkodie and Owusu (2016). In the long run, the interaction between the two variables does not have significant impact on CO_2_ emission. This further implies that the interaction between the two variables is environmentally sustainable in the short run.

For the third model, which shows the impact of the interaction between renewable energy sources and ICT on CO_2_ emissions, it is revealed that in Column 3 of Table 13, the CS-ARDL results show that in the short and long run, the interaction between renewable energy sources and ICT has a positive and significant impact on CO2 emissions. This indicate that a 1% increase in the interaction between renewable energy sources and ICT in the short and long run leads to 0.15% and 0.15% increase in CO_2_ emission, respectively. This implies that the interaction between the two variables contributes to the increase in CO_2_ emission both in the short and long run. This finding is not consistent with the studies conducted by Ozcan and Apergis (2018) for emerging countries, Salahuddin and Alam (2015) for Australia, Ibrahim and Waziri (2020) for SSA, and Mehrjo et al. (2022) for Iran. The differences in the findings could be due to various factors, such as differences in the time period studied, the theoretical framework used, econometrics techniques and other related factors. This is not as expected for the BRICS economy, and some possible reasons for the short- and long-run results could be that: (i) the increased renewable energy consumption of ICT infrastructures has not been sufficient to contribute to the reduction of CO_2_ emissions given that the energy use in the BRICS countries is mostly generated from fossil fuels/non-renewable sources, on which they heavily rely. (ii) Their production and disposal of ICT devices and equipment could have led to significant environmental damage and carbon emissions. This includes the energy used in the production of ICT devices, as well as the carbon emissions generated by the disposal of electronic/ICT devices. (iii) the possible poor/inadequate link between the manufacturing and transportation of renewable energy equipment and the concurrent use of ICT infrastructures. This is especially true if the manufacturing and transportation of renewable energy equipment, such as solar panels, wind turbines, etc., processes are not properly optimized with the use of ICT infrastructures to reduce carbon emissions (Lee et al. 2017; Godil et al. 2021; Khan et al. 2022a, b; Batool et al. 2022).

For the fourth model, which shows the institutional quality-ICT induced impact on CO_2_ emissions reveal that in Column 4 of Table 13, the CS-ARDL results show that in the short run, the interaction between the two variables has a negative and significant impact on CO_2_ emissions. This indicate that a 1% increase in the interaction between the two variables leads to -0.02% decrease in CO_2_ emission. This agrees with the findings of Cansino et al. (2019) Haldar and Sethi (2021) and Mahjabeen et al. (2020). While in the long run the interaction between the two variables does not have a significant impact on CO_2_ emission. This implies that the interaction between institutional quality and ICT contributes to the reduction of CO_2_ emission in the short run, while in the long run, it does not have a significant impact on CO_2_ emission. This further implies that the interaction between the two variables is environmentally sustainable in the short run.

Based on the results, the negative impact of the interaction between ICT and institutional quality on CO_2_ emissions in the short run is significant. This can be attributed to the fact that BRICS countries, with their growing economies, large populations, and increasing global political influence, have more advanced institutional development compared to many other developing countries. However, it is important to recognize that each BRICS country faces its own unique challenges and opportunities for development, with progress varying significantly within each nation. The success of policies related to industry, ICT, the environment, and renewable energy sources is heavily influenced by the effectiveness of domestic institutions. Without supportive institutions in the long term, the government's ability to enact and enforce policies in these sectors can be severely hampered. Therefore, it is crucial to implement and review strategies and initiatives that promote the six governance indicators used to compute institutional quality, to ensure ICT development and mitigate CO_2_ emissions for environmental sustainability in the BRICS countries. Additionally, BRICS governments should consider imposing long-term CO_2_ emission caps on the economic, ICT, and industrial sectors to keep environmental pollution levels under control. This is crucial because, in theory, ICT could promote environmental pollution through the production of machinery and devices, as well as the recycling of electronic waste. Furthermore, polluters are less likely to engage in pollution-causing behavior if they fear facing harsh punishment, which could take any form adopted by governments. Given that industrial production processes in BRICS still heavily rely on dirty energy, it is important for industrialists to consider integrating and promoting the use of ICT to achieve environmental sustainability.

### Robustness checks

As a means of testing the robustness of the CS-ARDL results, the study followed similar approaches adopted in recent literature (Wang 2007; Sharif et al. 2022) by using models that could account for cross-sectional dependence in the long run. In doing so, AMG and CCEMG estimation were applied to the variables under consideration. Results for both these tests are reported in Table 14. Upon reflection of the estimates provided in Table 14, we were able to establish that the signs for all coefficients for both the AMG and CCEMG for the respective control variables, were relatively consistent with those reported in the CS-ARDL estimations. The results of AMG and CCEMG for the interacted variables (i.e., the interaction between industrialization and ICT, renewable energy sources and ICT, institutional quality and ICT) are consistent with the long run results of CS-ARDL.

**Table 14:** Robustness test results using AMG and CCEMG estimators

	(1)	(2)	(3)	(4)	(5)	(6)	(7)	(8)
Variables	AMG 1	CCEMG 1	AMG 2	CCEMG 2	AMG 3	CCEMG 3	AMG 4	CCEMG 4
LIND	0.437**	0.461*	0.536	0.612***	0.484***	0.612***	0.268	0.519***
	(0.200)	(0.268)	(0.831)	(0.047)	(0.129)	(0.047)	(0.169)	(0.085)
LGDPC	0.635***	0.721***	0.704***	0.633***	0.769***	0.633***	0.458***	0.452***
	(0.121)	(0.238)	(0.219)	(0.219)	(0.192)	(0.219)	(0.135)	(0.160)
LICT	-0.068	-0.090**	-0.014	-0.012	0.003	-0.012	-0.039**	0.065
	(0.044)	(0.044)	(1.517)	(0.074)	(0.083)	(0.074)	(0.019)	(0.111)
RESS	-0.172*	-0.256**	-0.077*	-0.258	-0.294*	-0.351*	-0.501**	-0.434**
	(0.100)	(0.129)	(0.046)	(0.202)	(0.171)	(0.181)	(0.248)	(0.203)
INSQTY	0.008	0.005***	-0.016*	-0.009	-0.031	0.010***	-0.017	-0.003
	(0.012)	(0.001)	(0.009)	(0.013)	(0.032)	(0.003)	(0.028)	(0.022)
LIND*LICT			-0.003	-0.451				
			(0.421)	(0.276)				
RESS*LICT					0.104*	0.170*		
					(0.070)	(0.094)		
INSQTY*LICT							-0.011	-0.001
							(0.022)	(0.031)
Constant	-4.135***	1.290	-8.094**	0.149	-6.744***	2.277	-3.250***	-0.360
	(0.327)	2.505	(3.165)	4.870	(2.378)	2.087	(0.513)	2.135
Obs	91	91	91	91	91	91	91	91
Wald Chi^2^	38.06***	35.46***	16.85***	56.80***	36.22***	201.45***	4219.00***	50.16***

Standard errors in parentheses; *, ** and *** represent significance at the 1%, 5%, and 10% levels respectively. **Source:** Author’s Computations

## Policy implications

Based on the results, the policymakers and governments should utilize the levels of ICT development to gain insights into the risks associated with environmental degradation and promote sustainable values for environmental sustainability in BRICS. Governments should invest in BRICS's ICT, renewable energy sources, industrial and institutions to promote environmental sustainability. This can be achieved by enhancing positive behaviours and modifying behaviour in BRICS countries. In order to encourage and support BRICS countries to actively participate in the fight against environmental degradation/global warming, one of the challenges they may face is accessing the global ICT market.

To effectively reduce CO_2_ emissions through the use of renewable energy sources, it is imperative that BRICS countries prioritize and enhance the integration of ICT in the production processes of renewable energy products. Given the budgetary constraints faced by these nations, it is recommended that investments and resource allocations in the ICT and renewable energy sectors be pursued in collaboration with the private sector. Furthermore, BRICS governments should develop and implement comprehensive policies for ICT and renewable energy, outlining specific objectives and providing financial incentives for investments in these areas. This strategic approach will not only elevate the development of ICT and increase the share of renewable energy in the energy mix but will also contribute significantly to the achievement of Sustainable Development Goals 7 (Affordable and Clean Energy) and 9 (Industry, Innovation, and Infrastructure), thereby promoting environmental sustainability and fostering innovation within the BRICS nations.

The detrimental effect of the interplay between ICT and institutional quality on CO_2_ emissions highlights the need for policymakers to concentrate on bolstering institutions through pertinent policies. Such actions would aid in the adoption of technologies, ultimately paving the way for long-term environmental sustainability. Potential institutional policies to consider include enhancing transparency and accountability, improving regulatory frameworks, fostering public–private partnerships, and investing in education and training to build capacity for technology adoption. Furthermore, policies that strengthens institutions that facilitate technological property right protections in the renewable energy sources portfolio will be crucial for reducing CO_2_ emissions since it facilitates technological innovation.

Based on the CS-ARDL estimates, this study recommends that BRICS governments and policymakers can achieve environmental sustainability more quickly in the short term by utilizing ICT in conjunction with levels of industrialization and institutional quality. The core theoretical contribution of this study is that ICT could acts as an information sharing mechanism if proper measures are put in place by promoting industrialization and institutional quality in curbing CO_2_ emissions. The managerial implications of this study within the context of the industrial, renewable and institutional sectors are as follows, firstly, managers in the industrial sector can integrate ICT in the production process to reduce energy consumption, optimize resource usage, and minimize waste generation. By using ICT services/infrastructures industrial processes can be made more efficient, thereby reducing their carbon emissions. Secondly, managers in the institutional sector can use ICT to improve communication and collaboration among different levels of government and agencies. This can facilitate the implementation of policies and programs that promote environmental sustainability, such as the use of renewable energy sources, waste reduction, and conservation. In addition, the use of ICT can provide innovative solutions to help managers in these sectors reduce their negative environmental impact while improving their operations and profitability in BRICS economies.

This study advises caution in the long term as the interaction between ICT and renewable energy sources, industrialization, and institutional quality may not favour environmental quality. Although the renewable energy sources interaction with ICT may not yield immediate progress, strong measures need to be taken to ensure that short-term gains are not nullified. In conclusion, the study highlights the potential of ICT, renewable energy sources, industrialization, and institutional quality in achieving environmental sustainability in the BRICS countries, while recommending cautious measures in the long run to safeguard the progress made.

## Conclusion

As BRICS countries—Brazil, Russia, India, China, and South Africa—strive to achieve the United Nations Sustainable Development Goal 13, which is climate change mitigation, there is a growing need to harness ICT, renewable energy sources, levels of industrialization and institutional quality to achieve this goal. Therefore, it is crucial to explore the potential role that ICT development, renewable energy sources, industrialization, and institutional quality can play in promoting environmental sustainability in BRICS economy. The study covers the period from 2000 to 2021 and utilized a range of innovative econometric techniques, which are outlined in the third section of this study. This research is unique compared to previous literature because it examines the impact of the interaction between ICT and industrialization, ICT and renewable energy sources, ICT and institutional quality on CO_2_ emissions. This is because globally, regionally and economic bloc wise, the use of ICT services and infrastructures has permeated all sectors of the economy. Consequently, formulating integrated policies that are informed by the findings of this study has become critical in the BRICS context.

This study contributes to the literature by providing the following results. Firstly, a long-run equilibrium relationship was established among the variables, while the causality results reveal that there is a two-way causality between GDP per capita and CO_2_ emissions. While unidirectional causality runs from: CO_2_ emissions to industrialization; ICT to CO_2_ emissions; and renewable energy sources to CO_2_ emissions. Secondly, for the long run coefficient estimates, the results from the FMOLS and the DOLS reveal that ICT and industrialization, ICT and institutional quality significantly contribute to the reduction of CO_2_ emissions.

Thirdly, In Model 1, CS-ARDL results show a positive and significant impact of GDP per capita and industrialization on CO_2_ emissions, a negative and insignificant impact of institutional quality on CO_2_ emissions, and a positive and insignificant impact of ICT on CO_2_ emissions. In the remaining Models, CS-ARDL short run results indicate that the interaction between industrialization and ICT has a significant and negative impact on CO_2_ emissions, the interaction between renewable energy sources and ICT has a significant and positive impact on CO_2_ emissions, and the interaction between institutional quality and ICT has a significant and negative impact on CO_2_ emissions. In the long run, the interaction between industrialization and ICT has an insignificant and negative impact on CO_2_ emissions, the interaction between renewable energy sources and ICT has a significant and positive impact on CO_2_ emissions, and the interaction between institutional quality and ICT has an insignificant and negative impact on CO_2_ emissions. Lastly, our research is crucial for the strategic development of climate change policies designed to lessen the effects of climate change in BRICS, because ICT development interaction with renewable energy sources, industrialization and institutional quality must be taken seriously to ensure environmental sustainability.

Our study focuses on BRICS economies and utilizes a panel dataset covering the period from 2000 to 2021, primarily due to data limitations. Future research endeavors incorporating more extensive data that includes recently added countries to the BRICS and other regions, such as Europe & Central Asian (ECA), East & South Asia and the Pacific (ESAP), and the Americas, would provide valuable insights for governments and policymakers on leveraging ICT development, renewable energy, agriculture, industry, and institutional sectors to promote environmental sustainability. This is especially relevant as governments in these regions confront the challenge of achieving Sustainable Development Goal 13. Furthermore, future research should examine whether the established conclusions in this study withstand empirical scrutiny within country-specific or other economic bloc settings to enhance the current understanding of this study's research topic. This is essential for more country-specific policy implications.

## Data Availability

All data generated or analysed during this study are not included in this submission but can be made available upon reasonable request.

## Footnotes

1 Due to the governing rules, it is important for the reader to take note that we did not log variables with negative values.

2 Studies that have used these techniques include those of Saba & Ngepah (2019a, 2019b, 2019c, 2019d, 2022a, 2022b, 2022c, 2022d, 2023), Saba (2020a, 2020b, 2020c, 2021a, 2021b, 2023a, 2023b), Saba et al. (2023a, 2023b, 2024a, 2024b), Ngepah et al. (2024), Saba and Biyase (2022) and Saba and Pretorius (2024).
